# Ações de vigilância alimentar e nutricional no Brasil e em Portugal: uma análise documental comparativa

**DOI:** 10.1590/0102-311XPT189823

**Published:** 2024-09-16

**Authors:** Iolanda Karla Santana dos Santos, Andreia Oliveira, Sara Araújo da Silva, Wolney Lisboa Conde

**Affiliations:** 1 Faculdade de Saúde Pública, Universidade de São Paulo, São Paulo, Brasil.; 2 Fundação Universidade Federal do ABC, Santo André, Brasil.; 3 Instituto de Saúde Pública, Universidade do Porto, Porto, Portugal.; 4 Ministério da Saúde, Brasília, Brasil.; 5 Centro Universitário Euro-Americano, Brasília, Brasil.

**Keywords:** Vigilância Alimentar e Nutricional, Política Nutricional, Sistemas de Informação em Saúde, Promoção da Saúde, Inquéritos Populacionais, Food and Nutritional Surveillance, Nutrition Policy, Health Information Systems, Health Promotion, Demographic Surveys, Vigilancia Alimentaria y Nutricional, Política Nutricional, Sistemas de Información en Salud, Promoción de la Salud, Encuestas de Población

## Abstract

A vigilância alimentar e nutricional é fundamental para a formulação, a implementação, o monitoramento e a avaliação de políticas públicas de alimentação e nutrição. A análise comparativa da evolução das ações de vigilância alimentar e nutricional entre países permite o conhecimento de avanços, desafios e inovações para o planejamento de intervenções. O objetivo foi descrever e comparar as ações de vigilância alimentar e nutricional no Brasil e em Portugal, países com dimensões geográficas e perfis socioeconômicos distintos, mas com sistema de saúde universal em comum. Nós buscamos por documentos que descrevessem as ações de vigilância alimentar e nutricional e por potenciais fontes de dados de maneira manual em *websites* institucionais dos governos brasileiro e português. O estudo utilizou as recomendações da abordagem READ (59 documentos foram identificados para o Brasil e 29 para Portugal). No Brasil, as ações de vigilância alimentar e nutricional estão inseridas em políticas de saúde e nas condicionalidades dos programas de transferência condicionada de renda. As *Pesquisas de Orçamentos Familiares* e os inquéritos de saúde são utilizados simultaneamente no Sistema de Vigilância Alimentar e Nutricional (SISVAN). Em Portugal, as ações de vigilância alimentar e nutricional estão inseridas nas políticas de saúde, por meio do Programa Nacional e da Estratégia Integrada para a Promoção da Alimentação Saudável. Entre as fontes de dados identificadas estão a Balança Alimentar Portuguesa, os inquéritos de orçamentos familiares, de saúde e alimentares, além de iniciativas para o monitoramento do estado nutricional de crianças e adolescentes. Em ambos os países, as estratégias precisam ser aprimoradas em relação à regularidade do registro de dados, à harmonização dos indicadores do consumo alimentar e à disseminação das informações.

## Abstract (en)

Food and nutrition surveillance is fundamental to the formulation, implementation, monitoring, and evaluation of public policies on food and nutrition. The comparative analysis of the evolution of food and nutrition surveillance actions between countries makes it possible to learn about advances, challenges and innovations for the planning of interventions. This study aimed to describe and compare food and nutrition surveillance actions in Brazil and Portugal, countries with different geographical dimensions and socio-economic profiles, but with the common characteristic of having a universal healthcare system. We manually searched for documents describing food and nutrition surveillance actions and for potential data sources on the institutional websites of the Brazilian and Portuguese governments. The recommendations of the READ approach were used (59 documents were identified for Brazil and 29 for Portugal). In Brazil, food and nutrition surveillance actions are included in health policies and in the constraints of conditional cash transfer programs. The *Brazilian Households Budget Surveys* and health surveys are used simultaneously with the Brazilian National Food and Nutrition Surveillance System (SISVAN). In Portugal, food and nutrition surveillance actions are part of health policies, via the National Program and the Integrated Strategy for the Promotion of Healthy Eating. Among the data sources identified are the Portuguese Food Balance Sheet and household budget, health and food surveys, as well as initiatives to monitor the nutritional status of children and adolescents. In both countries, strategies need to be improved in terms of the regularity of data recording, the harmonization of food consumption indicators and the dissemination of information.

## Resumen

La vigilancia alimentaria y nutricional es fundamental para la formulación, implementación, monitoreo y evaluación de las políticas públicas de alimentación y nutrición. El análisis comparativo de la evolución de las acciones de vigilancia alimentaria y nutricional entre países permite el conocimiento de los avances, desafíos e innovaciones para la planificación de intervenciones. El objetivo fue describir y comparar las acciones de vigilancia alimentaria y nutricional en Brasil y en Portugal, países con diferentes dimensiones geográficas y perfiles socioeconómicos, pero con un sistema de salud universal en común. Buscamos documentos que describieran las acciones de vigilancia alimentaria y nutricional y posibles fuentes de datos manualmente en sitios *web* institucionales de los gobiernos de Brasil y Portugal. El estudio utilizó las recomendaciones del enfoque READ (se identificaron 59 documentos para Brasil y 29 para Portugal). En Brasil, las acciones de vigilancia alimentaria y nutricional están incluidas en políticas de salud y en las condicionalidades de los programas de transferencia condicionada de ingresos. Las *Encuestas de Presupuestos Familiares* y los estudios de salud se utilizan simultáneamente con el Sistema de Vigilancia Alimentaria y Nutricional (SISVAN). En Portugal, las acciones de vigilancia alimentaria y nutricional están incluidas en las políticas de salud, por medio del Programa Nacional y de la Estrategia Integrada para la Promoción de una Alimentación Saludable. Entre las fuentes de datos identificadas, se encuentran la Balanza Alimentaria Portuguesa, las encuestas de presupuestos familiares, de salud y alimentarias. Además de iniciativas para el monitoreo del estado nutricional de niños y adolescentes. En ambos países, es necesario mejorar las estrategias con relación a la regularidad del registro de datos, a la armonización de los indicadores de consumo de alimentos y a la difusión de la información.

## Introdução

De acordo com a Organização Mundial da Saúde (OMS), a vigilância alimentar e nutricional é um conceito multisetorial que engloba a vigilância de componentes desde os sistemas alimentares, acesso a alimentos, condições socioeconômicas até os desfechos em saúde ^1^ ^,^ ^2^. Considerando os componentes relacionados à saúde, a vigilância alimentar e nutricional tem como objetivo o monitoramento contínuo do consumo alimentar e do estado nutricional de determinada população ^3^ ^,^ ^4^. Dessa forma, espera-se que a vigilância alimentar e nutricional forneça informações para a formulação de políticas e para a reformulação do próprio sistema, estabelecendo um ciclo dinâmico de formulação, implementação e monitoramento de políticas públicas ^1^ ^,^ ^2^ ^,^ ^4^.

Nesse sentido, os objetivos da vigilância alimentar e nutricional são descrever o estado nutricional da população, fornecer informações sobre potenciais causas e fatores associados, promover decisões embasadas pelos gestores públicos, acompanhar o progresso para alcançar metas relacionadas à nutrição, permitir análises de tendências para previsões de cenários, avaliar a cobertura de serviços, monitorar programas e detectar o impacto de mudanças em políticas ^1^ ^,^ ^3^ ^,^ ^4^ ^,^ ^5^. Para a consecução desses objetivos, a vigilância alimentar e nutricional pode ser executada por meio de diferentes estratégias que variam em função do território, do problema e do público-alvo ^2^. De modo geral, as fontes de dados podem ser administrativas ou de inquéritos ^2^. As fontes administrativas têm como limite o fato de a representatividade frequentemente ser desconhecida ^2^ ^,^ ^4^. Ao mesmo tempo, oferecem ampla gama de informações, por exemplo, sistemas de informação em saúde e censos escolares que permitem inclusive a desagregação dos dados a territórios como vilas ou municípios ^2^ ^,^ ^4^. Outra vantagem é o uso desses dados administrativos na comparação de políticas públicas de alimentação e nutrição. Essa comparação permite a análise de diferenças e semelhanças entre as estratégias de vigilância alimentar e nutricional adotadas nos países ou regiões.

Os inquéritos, quando executados de maneira apropriada, produzem dados representativos da população e coletam um número significativo de variáveis, as quais são úteis para a análise dos fatores de risco e proteção para os agravos nutricionais ^2^. Os Estados Unidos são um exemplo na utilização de inquéritos para monitoramento nutricional de sua população. O *National Health and Nutrition Examination Survey* (NHANES) tem sido conduzido periodicamente desde 1971. Outros inquéritos também se destacam cobrindo diferentes fases da vida e grupos populacionais específicos ^6^. A realização de inquéritos com o objetivo de produzir informação em níveis territoriais menores é financeiramente onerosa ^2^. Alternativamente, é possível realizar inquéritos em pequena escala com repetição que utilizam métodos padronizados para coletar os dados em nível local em determinado período ^4^. Esse tipo de abordagem foi utilizado para monitorar o estado nutricional em situação de emergência no Sudão do Sul. Na abordagem, priorizou-se áreas para as quais não havia inquéritos recentes e nas quais o nível de insegurança alimentar e nutricional era elevado. Em experiências anteriores, observou-se que a situação nutricional em condições adversas pode se deteriorar rapidamente. Por isso, realizou-se mais de uma coleta de dados em cada área para avaliar a prevalência de desnutrição em diferentes momentos ^7^.

Analisando as estratégias utilizadas para a vigilância alimentar e nutricional, a escolha dos indicadores para monitoramento deve considerar as possíveis causas dos agravos nutricionais, a viabilidade da coleta nas condições locais (facilidade para mensurar o fenômeno, frequência e custo), a validade, a confiabilidade, a sensibilidade, a especificidade e a coerência com o contexto local ^1^ ^,^ ^4^. Além disso, os indicadores devem ser sensíveis para as mudanças críticas no estado nutricional da população e o monitoramento de tendências ^1^. Para avaliar mudanças em períodos curtos, a prevalência de magreza e de circunferência do braço pequena são bons indicadores de desnutrição aguda ^5^. Enquanto a prevalência de déficit de altura é um bom indicador para avaliar os cuidados em longo prazo. De modo geral, alterações nesse indicador são reflexos de mudanças estruturais ^5^. Outro aspecto desejável é que os países monitorem indicadores comparáveis (harmonizados) que sejam produzidos a partir de medidas similares e padronizadas ^3^.

A obtenção dos indicadores é fundamental para a realização da vigilância alimentar e nutricional, mas apenas a obtenção não garante a sua utilização pelos gestores públicos ^8^. Dessa forma, é necessário que as informações obtidas alcancem e influenciem os gestores públicos na tomada de decisões ^8^. Para isso, as informações devem ser apresentadas de maneira compreensível no momento oportuno e serem relevantes para auxiliar os gestores públicos em suas decisões ^4^ ^,^ ^8^. As informações também podem ser direcionadas a outros públicos, como agências financiadoras, pesquisadores e público geral, com diferentes níveis de desagregação e estratégias de disseminação ^4^ ^,^ ^8^ ^,^ ^9^. As forças sociais, políticas e econômicas podem influenciar a realização das ações necessárias para a formulação e a implementação de políticas ^8^ ^,^ ^9^. A disseminação dos dados potencializa ações de advocacia social dentro do setor público ou por organizações não governamentais e movimentos sociais fomentando a implementação de políticas para grupos vulneráveis ou populações em desvantagem ^4^ ^,^ ^9^ ^,^ ^10^.

A pesquisa documental contribui para a obtenção e organização de informações acerca das origens, dos fundamentos e das inovações das ações de vigilância alimentar e nutricional, bem como de seus avanços e desafios. A compreensão dos percursos históricos da evolução das ações de vigilância alimentar e nutricional em dois territórios que guardam características comuns como um sistema de saúde universal e particularidades como dimensões geográficas e perfis socioeconômicos distintos, contribui para a reflexão acerca do planejamento das ações de vigilância alimentar e nutricional frente a uma conjuntura epidemiológica desfavorável. O objetivo deste estudo foi descrever e comparar as ações de vigilância alimentar e nutricional no Brasil e em Portugal.

## Métodos

### Descrição dos casos

O Brasil, localizado na América do Sul, apresenta uma área territorial de 8.510.345,538km^2^, população de 203.062.512 pessoas (de acordo com o *Censo Demográfico* de 2022) ^11^ e Índice de Desenvolvimento Humano (IDH), em 2021, de 0,754 ^12^.

Em 2019, no Brasil, as principais causas de morte e incapacidades combinadas foram: (1) violência interpessoal; (2) doença isquêmica do coração; (3) desordens neonatais; (4) doença cerebrovascular; (5) diabetes; (6) acidente de trânsito; (7) dor lombar baixa; (8) infecções respiratórias inferiores; (9) cefaleias; e (10) transtornos de ansiedade ^13^. Em 2019, as mortes e incapacidades combinadas foram atribuídas aos seguintes fatores de risco: (1) elevado índice de massa corporal (IMC); (2) pressão arterial alta; (3) tabagismo; (4) glicemia de jejum aumentada; (5) fatores dietéticos; (6) consumo de bebidas alcoólicas; (7) má nutrição; (8) lipoproteína de baixa densidade (LDL) colesterol elevado; (9) disfunção renal; e (10) poluição do ar ^13^. No mesmo ano, 60,3% dos adultos apresentavam excesso de peso (pré-obesidade e obesidade combinadas), e 25,9% obesidade ^14^.

No Brasil, o Sistema de Vigilância Alimentar e Nutricional (SISVAN) é um sistema de informação da atenção primária à saúde (APS) ^15^. Desde a publicação da Política Nacional de Alimentação e Nutrição (PNAN) ^16^, o SISVAN consolidou-se como estratégia prioritária de vigilância alimentar e nutricional. Nós estimamos a cobertura nacional do estado nutricional do SISVAN com os dados disponibilizados no *website* openDataSUS ^17^. A cobertura nacional do consumo alimentar foi estimada a partir dos relatórios consolidados do SISVAN ^18^. Nós utilizamos como denominador a população total do censo de 2022 ^11^. A cobertura nacional do estado nutricional atingiu 22,6% em 2022, enquanto a cobertura do consumo alimentar atingiu 1,25% em 2021.

Já Portugal, localizado na Europa, apresenta área territorial de 92.225,20km^2^, população estimada, em 2022, de 10.467.366 pessoas ^19^ e IDH de 0,866 em 2021 ^12^.

Em 2019, em Portugal, as principais causas de morte e incapacidades combinadas foram: (1) doença cerebrovascular; (2) doença isquêmica do coração; (3) dor lombar baixa; (4) diabetes; (5) doença pulmonar obstrutiva crônica (DPOC); (6) transtornos depressivos; (7) câncer de pulmão; (8) infecções respiratórias inferiores; (9) câncer colorretal; e (10) doença de Alzheimer ^20^. Nesse mesmo ano, as mortes e incapacidades combinadas foram atribuídas aos seguintes fatores de risco: (1) glicemia de jejum aumentada; (2) tabagismo; (3) pressão arterial alta; (4) elevado IMC; (5) fatores dietéticos; (6) consumo de bebidas alcoólicas; (7) LDL colesterol elevado; (8) disfunção renal; (9) riscos ocupacionais; e (10) temperatura não ideal ^20^. A obesidade também é um problema de saúde pública em Portugal, sendo que 58,1% da população adulta portuguesa apresentava excesso de peso (pré-obesidade e obesidade combinadas), e 21,6% apresentava obesidade, de acordo com dados objetivamente medidos no *Inquérito Alimentar Nacional e de Atividade Física* (IAN-AF) 2015-2016 ^21^.

Desde 2007, a *Iniciativa de Vigilância da Obesidade Infantil* (*Childhood Obesity Surveillance Initiative* - COSI) é promovida pela OMS em parceria com países da Europa com o objetivo de realizar o monitoramento das tendências de excesso de peso e obesidade em crianças. Em Portugal, a sexta ronda do COSI foi conduzida no ano letivo de 2021-2022. O COSI é baseado na estratégia de amostras transversais repetidas de crianças inscritas no 1º ciclo do Ensino Básico, que corresponde ao 1º e 2º anos escolares e abrange crianças dos 6 até 9 anos de idade incompletos. A cada três rondas, executa-se uma nova amostragem na qual as escolas são denominadas como Rede de Escolas Sentinelas. A participação das escolas foi de 98,3% em 2016, 99,1% em 2019 e de 96,6% em 2022. Em 2022, a amostra do COSI alcançou 6.205 crianças, 77,4% do esperado. O número de respostas ao questionário foi de 4.791 famílias (59,6%) ^22^.

### Estratégia de busca

Nós buscamos por documentos que descrevessem as ações de vigilância alimentar e nutricional e por potenciais fontes de dados para vigilância alimentar e nutricional no Brasil e em Portugal. A busca foi realizada de maneira manual por um dos autores em *websites* institucionais dos governos brasileiro e português entre outubro de 2021 e fevereiro de 2022. Também foram incluídos documentos publicados após o período de busca pelos governos brasileiro e português. As listas de referências dos documentos foram consideradas para a identificação de documentos adicionais. A pesquisa foi refinada e expandida em resposta aos resultados das pesquisas anteriores e dos documentos identificados de maneira iterativa. Não houve restrições em relação ao idioma e ao ano de publicação.

### Critérios de elegibilidade

Nesta análise foram incluídos manuais técnicos, legislações, relatórios, protocolos e políticas que abordassem ações de vigilância alimentar e nutricional. As fontes de dados incluídas deveriam apresentar pelo menos um dos seguintes tipos de informação: antropometria, consumo alimentar, disponibilidade alimentar ou segurança alimentar e nutricional.

### Análise documental

Neste estudo, foram seguidas as recomendações da abordagem READ (*Ready materials, Extract data, Analyse data, Distil*, em tradução livre - ler os documentos, extrair os dados, analisar os dados e depurar) para a análise documental ^23^.

### Extração dos dados

Os documentos foram lidos e os dados foram extraídos por um dos autores e conferidos por outro autor. Os itens identificados para cada país foram organizados em dois grupos: (1) documentos; (2) potenciais fontes de dados para vigilância alimentar e nutricional. Do primeiro grupo foram extraídas as informações: título, autores, colaboradores, ano de publicação, tipo de documento, editora ou título do periódico, fonte, palavras-chave, público-alvo e síntese do documento. Já do segundo grupo foram extraídas as informações: identificação, agência coordenadora, parceiros, metodologia de coleta de dados, ano ou frequência de coleta de dados, abrangência, tamanho de amostra, seleção dos respondentes, objetivos, antropometria (sim ou não), consumo alimentar (sim ou não), disponibilidade alimentar (sim ou não), segurança alimentar e nutricional (sim ou não), divulgação dos resultados e fonte. Neste estudo, foram consideradas como fontes de dados administrativos as provenientes de sistemas de informação em saúde.

De modo a realizar uma análise abrangente e comparada das ações de vigilância alimentar e nutricional no Brasil e em Portugal foram adotadas as categorias: (1) fontes de dados; (2) indicadores; (3) disseminação dos dados; e (4) sustentabilidade. Os aspectos conceituais das categorias foram descritos na introdução deste artigo.

## Resultados

**Figura 1:**
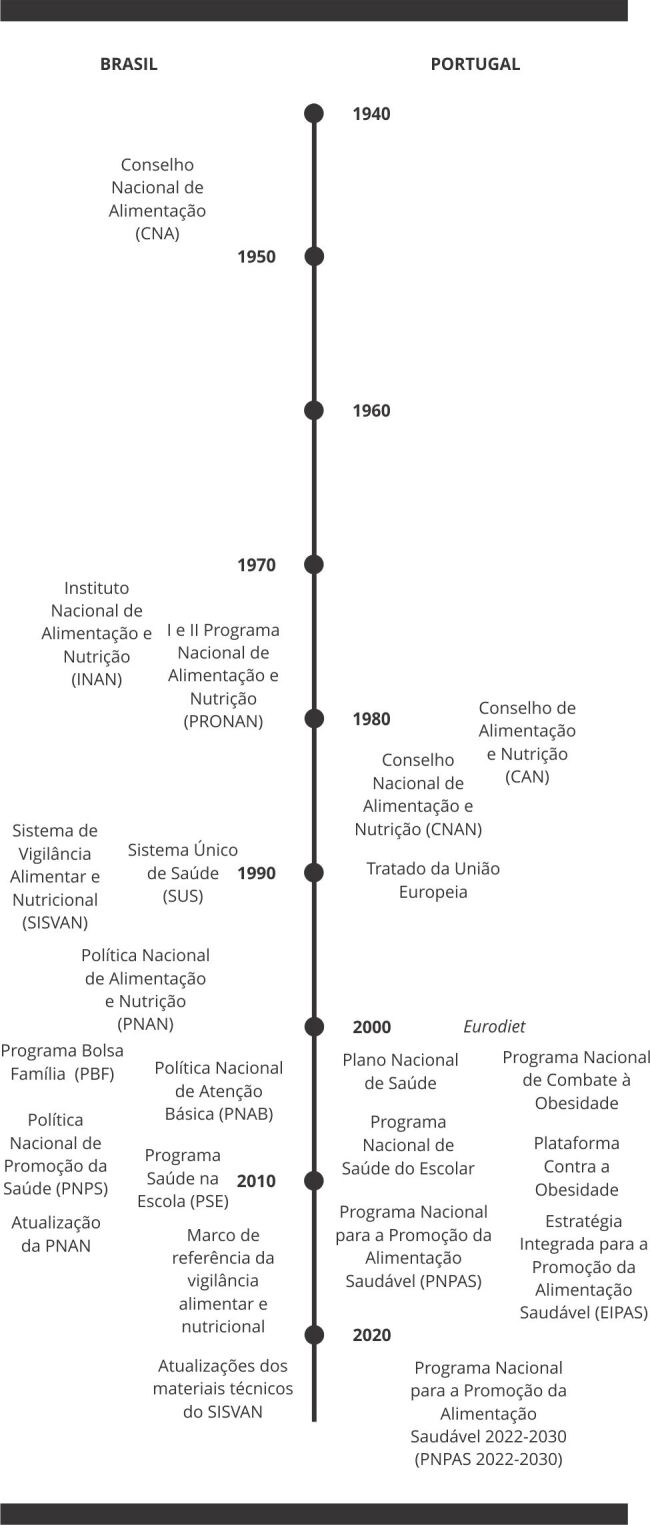
Visão geral das ações, programas e políticas relacionadas à vigilância alimentar e nutricional no Brasil (1945 a 2022) e em Portugal (1980 a 2022) ao longo do tempo.

Na Figura 1 são apresentados, de maneira esquemática, os principais marcos nas políticas de alimentação e nutrição no Brasil e em Portugal.

Para o Brasil, foram identificados 59 documentos distribuídos nos tipos legislação, política, relatório e manual (Material Suplementar - Quadro S1. https://cadernos.ensp.fiocruz.br/static//arquivo/supl-e00189823_6963.pdf). Entre os marcos temporais, destaca-se o ano de 1973 com o decreto de instituição do 1º Programa Nacional de Alimentação e Nutrição (PRONAN) ^24^ que abordava a vigilância do estado nutricional, a recuperação da desnutrição e orientou a execução de estudos e pesquisas, como o *Estudo Nacional da Despesa Familiar* (ENDEF) 1974-1975 ^25^. Em 1976, o 2º PRONAN tinha como finalidade a redução da pobreza absoluta e apoio ao pequeno produtor agrícola ^26^. Outro marco relevante foi a criação do SISVAN em 1990 por meio da *Portaria nº 1.156* no Ministério da Saúde ^27^, e consolidada na Lei Orgânica da Saúde ^28^. No Brasil, dois aspectos demandam e, ao mesmo tempo, promovem o fortalecimento da vigilância alimentar e nutricional. O primeiro deles é o conjunto das políticas do setor saúde: PNAN ^16^, Política Nacional de Atenção Básica (PNAB) ^29^ e Política Nacional de Promoção da Saúde (PNPS) ^30^. O segundo é relativo ao acompanhamento das condicionalidades de saúde em programas de transferência condicionada de renda ^31^.

No Quadro 1, são listadas as fontes de dados identificadas para a utilização na vigilância alimentar e nutricional. O Brasil adota o conceito ampliado de vigilância alimentar e nutricional que permite o uso de diferentes estratégias de vigilância epidemiológica a partir de dados administrativos obtidos durante os atendimentos na rotina dos serviços de saúde do Sistema Único de Saúde (SUS) ou de estudos e inquéritos populacionais ^15^. Os dados da fonte administrativa são compilados no SISVAN, a partir de suas interfaces com o Sistema de Gestão do Programa Bolsa Família na saúde e da estratégia e-SUS APS. Até 2004, o seu primeiro manual técnico abordava basicamente o acompanhamento do estado nutricional por meio da antropometria ^32^. A avaliação do consumo alimentar foi estabelecida em 2008 nos protocolos do SISVAN ^33^. Em 2015, o *Marco de Referência da Vigilância Alimentar e Nutricional na Atenção Básica* ^15^ posiciona os profissionais de saúde para a postura de vigilância no ciclo de gestão e produção do cuidado. Nesse mesmo ano, é publicado o documento de *Orientações para Avaliação de Marcadores de Consumo Alimentar na Atenção Básica* ^34^, no qual os formulários de marcadores do consumo alimentar foram atualizados em consonância com o *Guia Alimentar para a População Brasileira* ^35^. Em 2022, o *Guia para a Organização da Vigilância Alimentar e Nutricional na Atenção Primária à Saúde* ^36^ foi lançado com o objetivo de orientar gestores e profissionais na realização das ações de vigilância alimentar e nutricional. No SISVAN, é possível obter relatórios públicos do estado nutricional a partir de 2008 e dos marcadores do consumo alimentar a partir de 2015 da população, de todas as fases ou evento de vida, atendida na APS.

**Quadro 1:** Potenciais fontes de dados para a vigilância alimentar e nutricional no Brasil (1974 a 2022) e em Portugal (1963 a 2022), organizadas cronologicamente.

PERÍODO	BRASIL	PORTUGAL
1960-1990 (30 anos)	*Estudo Nacional da Despesa Familiar* - ENDEF 1974-1975 *Pesquisa Nacional sobre Saúde Materno-Infantil e Planejamento Familiar* - PNSMIPF 1986 *Pesquisa de Orçamentos Familiares* - POF 1987-1988 *Pesquisa Nacional sobre Saúde e Nutrição* - PNSN 1989	Balança Alimentar Portuguesa 1963-1975, 1980-1992 (+1990-1997, 1990-2003, 2003-2008, 2008-2012, 2012-2016, 2016-2020) *Inquérito às Receitas e Despesas Familiares* 1967-1968 *Inquérito Alimentar Nacional* 1980 *Inquérito às Receitas e Despesas Familiares* 1980-1981 *Inquérito Nacional de Saúde* - INS 1987 *Inquérito aos Orçamentos Familiares* 1989-1990
1991-2011 (20 anos)	*Pesquisa sobre Saúde Familiar no Nordeste Brasil* - PSFNe 1991 *Pesquisa Nacional sobre Demografia e Saúde* - PNDS 1996 *Pesquisa sobre Padrões de Vida* - PPV 1996-1997 *Estudo Multicêntrico sobre Consumo Alimentar* 1997 *I Pesquisa de Prevalência de Aleitamento Materno nas Capitais Brasileiras e Distrito Federal* 1999 *Pesquisa de Orçamentos Familiares* - POF 2002-2003 *Pesquisa Nacional por Amostra de Domicílios*: Segurança Alimentar - PNAD 2004 Chamada nutricional de crianças menores de 5 anos de idade residentes no semi-árido e assentamentos da Região Nordeste e do Norte de Minas Gerais 2005 Chamada nutricional para crianças menores de cinco anos de idade no Estado do Amazonas 2006 Chamada nutricional quilombola 2006 *Pesquisa Nacional de Demografia e Saúde da Criança e da Mulher* - PNDS 2006 *Vigilância de Fatores de Risco e Proteção para Doenças Crônicas por Inquérito Telefônico* - Vigitel 2006, 2007, 2008, 2009, 2010, 2011 (+2012, 2013, 2014, 2015, 2016, 2017, 2018, 2019, 2020, 2021, 2022) Chamada nutricional da Região Norte 2007 *II Pesquisa de Prevalência de Aleitamento Materno nas Capitais Brasileiras e Distrito Federal* 2008 *I Inquérito Nacional de Saúde e Nutrição dos Povos Indígenas* 2008-2009 *Pesquisa de Orçamentos Familiares* - POF 2008-2009 Sistema de Vigilância Alimentar e Nutricional - SISVAN 2008, 2009, 2010, 2011 (+2012, 2013, 2014, 2015, 2016, 2017, 2018, 2019, 2020, 2021, 2022) *Pesquisa Nacional de Saúde do Escolar* - PeNSE 2009 *Pesquisa Nacional por Amostra de Domicílios*: segurança alimentar - PNAD 2009	Balança Alimentar Portuguesa 1990-1997, 1990-2003, 2003-2008, 2008-2012 (+2012-2016, 2016-2020) *Inquérito aos Orçamentos Familiares* 1994-1995 *Inquérito Nacional de Saúde* - INS 1995-1996 *Health Behavior in School Children* - HBSC 1997-1998, 2001-2002, 2005-2006, 2009-2010 (+2013-2014, 2017-2018) *Inquérito Nacional de Saúde* - INS 1998-1999 *Inquérito aos Orçamentos Familiares* 2000 *Inquérito às Despesas das Famílias* 2005-2006 *Inquérito Nacional de Saúde* - INS 2005-2006 *Childhood Obesity Surveillance Initiative* - COSI 2008, 2010 (+2013, 2016, 2019, 2022) *Inquérito às Despesas das Famílias* 2010-2011 INFOFAMÍLIA 2011-2014
2012-2022 (10 anos)	*Vigilância de Fatores de Risco e Proteção para Doenças Crônicas por Inquérito Telefônico* - Vigitel 2012, 2013, 2014, 2015, 2016, 2017, 2018, 2019, 2020, 2021, 2022 Sistema de Vigilância Alimentar e Nutricional - SISVAN 2012, 2013, 2014, 2015, 2016, 2017, 2018, 2019, 2020, 2021, 2022 *Pesquisa Nacional de Saúde do Escolar* - PeNSE 2012 *Pesquisa Nacional de Saúde* - PNS 2013 *Pesquisa Nacional por Amostra de Domicílios*: segurança alimentar - PNAD 2013 *Estudo de Riscos Cardiovasculares em Adolescentes* - ERICA 2013-2014 *Pesquisa Nacional de Saúde do Escolar* - PeNSE 2015 *Pesquisa de Orçamentos Familiares* - POF 2017-2018 *Estudo Nacional de Alimentação e Nutrição Infantil* - ENANI 2019 *Pesquisa Nacional de Saúde* - PNS 2019 *Pesquisa Nacional de Saúde do Escolar* - PeNSE 2019	INFOFAMÍLIA 2011-2014 Balança Alimentar Portuguesa 2012-2016, 2016-2020 *Health Behavior in School Children* - HBSC 2013-2014, 2017-2018 *Childhood Obesity Surveillance Initiative* - COSI 2013, 2016, 2019, 2022 *Inquérito Nacional de Saúde* - INS 2014 *1º Inquérito Nacional de Saúde com Exame Físico* - INSEF 2015 *Inquérito Alimentar Nacional e de Atividade Física* - IAN-AF 2015-2016 *Inquérito às Despesas das Famílias* 2015-2016 *Inquérito Nacional de Saúde* - INS 2019

Além do SISVAN, desde 2006 é realizada anualmente a *Vigilância de Fatores de Risco e Proteção para Doenças Crônicas por Inquérito Telefônico* (Vigitel) nas capitais dos 26 estados brasileiros e no Distrito Federal em indivíduos adultos (≥ 18 anos) ^37^. Entre o conjunto de inquéritos que coletam informações de antropometria e alimentação e que são utilizados como fontes de dados na vigilância alimentar e nutricional, estão as pesquisas de orçamentos familiares, os inquéritos de saúde materno-infantil, os inquéritos de saúde escolar e os inquéritos de saúde e nutrição que compreendem objetivos amplos ^38^ ^,^ ^39^. No Brasil, também merecem destaque a realização de inquéritos para a análise de eventos específicos, como a amamentação, nas *Pesquisas de Prevalência de Aleitamento Materno nas Capitais Brasileiras* ^40^ e a insegurança alimentar, incluída na *Pesquisa Nacional por Amostra de Domicílios* (PNAD) ^41^, e a realização de estudos com populações específicas, como o *1º Inquérito Nacional de Saúde e Nutrição dos Povos Indígenas* realizado em 2008-2009 ^42^ (Material Suplementar - Quadro S2. https://cadernos.ensp.fiocruz.br/static//arquivo/supl-e00189823_6963.pdf).

Para Portugal, foram identificados 29 documentos distribuídos nos tipos legislação, política, plano de ação, relatório e outros (Material Suplementar - Quadro S3. https://cadernos.ensp.fiocruz.br/static//arquivo/supl-e00189823_6963.pdf). Em termos temporais, o documento inicial da análise é o *Decreto-Lei nº 265* ^43^ de 1980 que criou o Conselho de Alimentação e Nutrição (CAN) substituído em 1984 pelo Conselho Nacional de Alimentação e Nutrição (CNAN) ^44^. O CAN tinha como objetivo a formulação dos princípios orientadores de uma política de alimentação e nutrição para Portugal ^43^, que foi concretizada em 2012 com o Programa Nacional para a Promoção da Alimentação Saudável (PNPAS) ^45^. Esse programa enquadra-se num dos 11 programas de saúde prioritários do Plano Nacional de Saúde (Despacho nº 6.041/2016) ^46^. O PNPAS visa promover o estado de saúde da população portuguesa, atuando num dos seus principais determinantes: a alimentação ^45^. O PNPAS apresenta cinco objetivos: (1) aumentar o conhecimento sobre o consumo alimentar da população portuguesa, seus determinantes e suas consequências; (2) modificar a disponibilidade de certos alimentos, especialmente no ambiente escolar, de trabalho e em espaços públicos; (3) informar e habilitar para a compra, a produção e o armazenamento de alimentos saudáveis; (4) identificar e promover ações transversais que incentivem o consumo de alimentos saudáveis de forma articulada e integrada com outros setores; e (5) melhorar o modo de atuação dos diferentes profissionais que, pela natureza de sua atividade, possam influenciar conhecimentos, atitudes e comportamentos na alimentação individual e/ou em espaços coletivos ^45^. A partir da adesão de Portugal à União Europeia, as políticas de saúde sofreram influência direta e/ou indireta dos acordos realizados pelos Estados Membros do bloco, assim como também do Escritório Regional para a Europa da OMS ^47^.

Portugal não apresenta marcos legislativos para a incorporação da vigilância alimentar e nutricional em sua política nacional de saúde. Por isso, ações que podem ser enquadradas como vigilância são promovidas para a realização do diagnóstico nutricional da população, para o planejamento em saúde e para o monitoramento das metas pactuadas. Dessa forma, as ações de vigilância alimentar e nutricional estão inseridas nas políticas do setor saúde como o *Programa Nacional de Saúde Escolar* (PNSE) ^48^, a *Plataforma Contra a Obesidade* 2007-2011 ^49^, o PNPAS ^45^ e a Estratégia Integrada para a Promoção da Alimentação Saudável (EIPAS) ^50^. Esta última é uma estratégia intersetorial que apresenta um conjunto de 51 medidas de intervenção para promover a alimentação saudável dos portugueses, acordadas por sete Ministérios diferentes, sob a coordenação da Direção-Geral da Saúde ^51^. As 51 medidas de intervenção são divididas em quatro eixos: (1) modificar a disponibilidade de alimentos em determinados espaços e promover a reformulação de determinadas categorias de alimentos; (2) melhorar a qualidade e a acessibilidade da informação disponível ao consumidor; (3) promover e desenvolver a literacia e a autonomia para a realização de escolhas saudáveis; e (4) impulsionar a inovação e o empreendedorismo na promoção da alimentação saudável ^51^.

Em 2022, as novas linhas estratégicas do PNPAS para o período 2022-2030 elaboradas de acordo com o Plano Nacional de Saúde 2021-2030 foram divulgadas em documento de consulta pública. A elaboração da proposta foi baseada em evidências científicas e em modelos de planejamento estratégico, participativo e colaborativo em saúde ^52^. A situação atual das práticas alimentares e o estado nutricional da população portuguesa reforçam a importância da intervenção em nível do sistema de saúde. A proposta do PNPAS 2022-2030 ^52^ é um conjunto integrado de ações em três níveis: intervenção em nível ambiental, em nível individual e em nível do sistema de saúde. O documento apresenta sete valores e princípios, entre os quais o de evolução e adaptabilidade destacam o papel-chave do monitoramento da situação alimentar e nutricional para ajustar e adaptar as ações visando à melhoria da saúde da população portuguesa ^52^.

Na atualização da estratégia do PNPAS ^52^, foram estabelecidos três eixos nucleares e dois eixos de intervenção transversal. Os eixos nucleares são: (1) proteger e apoiar por meio da criação de ambientes alimentares saudáveis; (2) informar e capacitar cidadãos aptos a realizarem escolhas alimentares saudáveis; e (3) identificar e cuidar pela promoção da alimentação saudável no sistema de saúde e no acesso aos cuidados de saúde e à atenção nutricional. Os eixos transversais são: (4) monitorar e avaliar por meio de um sistema de informação de vigilância do consumo alimentar, estado nutricional e da insegurança alimentar; e (5) integrar e articular para uma ação que coloque a alimentação saudável em todas as políticas e que envolva toda a sociedade.

Entre as fontes disponíveis para a vigilância alimentar e nutricional (Material Suplementar - Quadro S4. https://cadernos.ensp.fiocruz.br/static//arquivo/supl-e00189823_6963.pdf), destaca-se o trabalho realizado pelo Instituto Nacional de Estatística português (INE) com os registros da Balança Alimentar Portuguesa, cujo primeiro relatório identificado apresenta resultados para o período de 1963-1975 ^53^. Considerando a categoria disponibilidade alimentar, outra fonte de dados são os inquéritos de orçamentos familiares também conduzidos pelo INE, sendo que a sua primeira edição foi em 1967-1968 ^54^. Em 1980, foi realizado o *1º Inquérito Alimentar Nacional* sob responsabilidade do Centro de Estudos de Nutrição do Instituto Nacional de Saúde Doutor Ricardo Jorge ^55^. O 2º inquérito, o IAN-AF, foi realizado apenas em 2015-2016 e conduzido por um consórcio, incluindo, em sua maioria, instituições de Ensino Superior com financiamento do Espaço Econômico Europeu, no âmbito de Iniciativas em Saúde Pública ^55^. Além desses, foram realizados seis inquéritos nacionais de saúde no total ^56^. Em uma abordagem clássica de vigilância alimentar e nutricional, destacam-se dois projetos conduzidos no ambiente escolar em parceria com a OMS, o *Comportamento de Saúde em Crianças em Idade Escolar* (*Health Behavior in School Children -* HBSC) ^57^ com adolescentes de 11, 13 e 15 anos, realizado desde 1997 e 1998, e o COSI com crianças em idade escolar do 1º ciclo do Ensino Básico (6 a 9 anos incompletos) ^58^.

No Quadro 2, são apresentadas as características da vigilância alimentar e nutricional em cada país. Em relação aos indicadores antropométricos do estado nutricional, os dois países utilizam os indicadores recomendados pela OMS ^59^ ^,^ ^60^ ^,^ ^61^. Os indicadores do consumo alimentar variam de acordo com a metodologia utilizada originalmente na fonte dos dados. De modo geral, o monitoramento dos indicadores busca mensurar comportamentos alimentares já identificados previamente como fatores de risco ou proteção para agravos nutricionais e/ou doenças crônicas, consumo abusivo de bebidas alcoólicas e consumo regular de frutas, legumes e verduras ^55^ ^,^ ^62^. Relativamente à disseminação dos resultados, nos dois países os dados dos inquéritos são difundidos preferencialmente por meio de relatórios técnicos e artigos científicos.

**Quadro 2:** Comparação das características da vigilância alimentar e nutricional entre Brasil e Portugal.

CARACTERÍSTICAS	BRASIL	PORTUGAL
Estratégias de vigilância alimentar e nutricional	Sistema de Vigilância Alimentar e Nutricional (SISVAN), com objetivos definidos	Vigilância alimentar e nutricional realizada com dados disponíveis
Fontes de dados	SISVAN e inquéritos populacionais	*Childhood Obesity Surveillance Initiative* (COSI) e inquéritos populacionais
Indicadores antropométricos do estado nutricional	Na população pediátrica, o indicador de déficit de altura e os indicadores de variação de massa corporal, como o déficit de peso, excesso de peso e obesidade, baseados em curvas de crescimento; e para a população adulta, os indicadores de excesso de peso e obesidade a partir do índice de massa corporal (IMC)	Na população pediátrica, o indicador de déficit de altura e os indicadores de variação de massa corporal, como o déficit de peso, excesso de peso e obesidade, baseados em curvas de crescimento; e para a população adulta, os indicadores de excesso de peso e obesidade a partir do IMC
Indicadores do consumo alimentar	Diferentes indicadores a depender das fontes de dados	Diferentes indicadores a depender das fontes de dados
Disseminação	*Site* com relatórios públicos do SISVAN, relatórios técnicos e artigos científicos	Relatórios técnicos e artigos científicos

No Brasil, a vigilância alimentar e nutricional está presente no art. 6, item 4, da *Lei nº 8.080/1990* ^28^ e a sua operacionalização ocorre como uma das diretrizes da PNAN ^16^. Portugal não possui uma regulamentação específica sobre a vigilância alimentar e nutricional e, apesar disso, tem realizado esforços como a implementação do COSI ^58^ e ações para o enfrentamento dos agravos relacionados à má nutrição por meio do PNPAS ^63^ e da EIPAS ^51^.

## Discussão

A vigilância alimentar e nutricional no Brasil e em Portugal compartilham as seguintes características: adoção de indicadores antropométricos baseados em curva de referência para a população pediátrica, classificação do estado nutricional pelo IMC para a população adulta, e avaliação do consumo alimentar por meio de indicadores relacionados a desfechos em saúde. No entanto, divergem em alguns aspectos. No Brasil, a vigilância alimentar e nutricional é realizada com dados de um sistema institucionalmente estabelecido e dados de inquéritos populacionais. Já em Portugal, a vigilância alimentar e nutricional é realizada principalmente com dados de inquéritos populacionais. Além disso, os países diferem na forma e nas estratégias de inserção das ações de vigilância alimentar e nutricional nas políticas de saúde.

### Fontes de dados

No Brasil, o sistema de vigilância alimentar e nutricional é definido e estruturado para atender aos objetivos de monitoramento contínuo do consumo alimentar e do estado nutricional da população atendida nos serviços da APS ^15^. A utilização dos dados do sistema na gestão de programas e políticas é direta. Por exemplo, os dados do SISVAN foram utilizados na Agenda para Intensificação da Atenção à Desnutrição Infantil (ANDI) em 2013 ^64^ ^,^ ^65^, e, no momento, são utilizados no monitoramento da Estratégia Nacional para Prevenção e Atenção à Obesidade Infantil (Proteja) ^66^. Em Portugal, as ações de VAN estão inseridas nas políticas de saúde. Essas ações foram fortalecidas no âmbito do PNPAS ^45^, no conjunto de metas e ações do programa em seus múltiplos eixos e com a utilização de dados produzidos por instituições acadêmicas, como o IAN-AF 2015-2016 ^55^. A EIPAS também é uma estratégia que tem fortalecido a vigilância no país, na medida em que demanda dados para a avaliação do cumprimento de suas metas ^67^.

As fontes de dados identificadas para a vigilância alimentar e nutricional foram variadas, como balança alimentar ^53^, disponibilidade domiciliar de alimentos ^28^ ^,^ ^54^, consumo alimentar individual ^55^, segurança alimentar e nutricional ^41^, avaliação antropométrica do estado nutricional ^68^ e avaliação bioquímica do estado nutricional ^69^. Essas fontes apresentam diferentes dimensões do estado nutricional e do consumo alimentar populacional. No Brasil, destaca-se o SISVAN que é um sistema de informação em saúde (SIS) com o papel de consolidar os registros de peso, altura e marcadores do consumo alimentar da população atendida na APS ^15^. Por se caracterizar como SIS da APS, no qual a inserção de dados varia em função das demandas dos serviços, os dados disponíveis no SISVAN não são representativos da população brasileira. Ainda que haja essa limitação, haveria estratégias analíticas para contorná-la, por exemplo, o cálculo de pesos pós-estratificação como já utilizados no Vigitel ^62^. Por outro lado, é razoável inferir que os dados disponíveis no SISVAN representam a população de maior vulnerabilidade numa proporção superior àquela que ocorre em cada território. Uma das bases para essa inferência está na obrigatoriedade do monitoramento do estado nutricional para aquelas famílias titulares do direito aos programas de transferência condicionada de renda. No Brasil há, ainda, outros sistemas informatizados para o monitoramento do estado nutricional de populações socialmente vulneráveis estabelecidos localmente. Por exemplo, o sistema Programa de Alimentação e Nutrição no qual são inseridos os dados antropométricos das crianças cadastradas no Projeto Estadual do Leite (Vivaleite) do Estado de São Paulo ^70^. O Vivaleite é uma iniciativa intersetorial e interinstitucional que tem como titulares de direito crianças de 6 meses até 6 anos e pessoas com idade superior a 60 anos ^71^ ^,^ ^72^.

Na perspectiva de uso simultâneo de diferentes fontes de dados, no Brasil estão disponíveis inquéritos populacionais representativos como a *Pesquisa Nacional de Demografia e Saúde da Criança e da Mulher* (PNDS) ^69^, realizada em 2006, e a *Pesquisa Nacional de Saúde* (PNS), realizada em 2013 ^39^ e em 2019 ^73^. Em Portugal, duas fontes de dados destacam-se em termos de vigilância alimentar e nutricional que são o HBSC ^57^ e o COSI ^58^, ambos realizados em parceria com a OMS. Adicionalmente, o INE tem elaborado relatórios frequentes atualizando a Balança Alimentar Portuguesa, inclusive comparando os dados observados com as recomendações para uma alimentação saudável dos portugueses e com os princípios preconizados pela dieta mediterrânea ^74^.

### Indicadores

O uso de diferentes fontes de dados contribui para o acompanhamento dos indicadores selecionados. No Brasil e em Portugal utilizam-se os mesmos indicadores antropométricos do estado nutricional. No Brasil, os marcadores do consumo alimentar do SISVAN são padronizados para a utilização na APS ^34^. Em relação aos inquéritos, equipes do Vigitel ^62^, PNS ^73^ e *Pesquisa Nacional de Saúde do Escolar* (PeNSE) ^75^ têm realizado tentativas de uniformização interna para a coleta desses indicadores para o monitoramento das tendências do consumo alimentar. No âmbito da União Europeia, está descrita a necessidade de harmonização dos indicadores da vigilância alimentar e nutricional entre os países do bloco há pelo menos 20 anos ^76^. Tanto no *site* do SISVAN (https://sisaps.saude.gov.br/sisvan/) quanto no do IAN-AF 2015-2016 (https://ian-af.up.pt/consulta-de-dados), é possível acessar indicadores do estado nutricional e do consumo alimentar da população brasileira e portuguesa, respectivamente.

Conforme mencionado anteriormente, a harmonização dos indicadores é importante internamente para o acompanhamento das tendências temporais e externamente para a comparação entre os países. Além do monitoramento das políticas públicas nacionais, o monitoramento por meio das ações de vigilância alimentar e nutricional é fundamental para o acompanhamento de metas globais. Como exemplo, citamos o objetivo 2 dos Objetivos de Desenvolvimento Sustentável (ODS) ^77^: “*erradicar a fome, alcançar a segurança alimentar, melhorar a nutrição e promover a agricultura sustentável*”. E a seguinte meta relacionada a esse objetivo: “*até 2030, acabar com todas as formas de má-nutrição, incluindo atingir, até 2025, as metas acordadas internacionalmente sobre nanismo e caquexia em crianças menores de cinco anos de idade, e atender às necessidades nutricionais dos adolescentes, mulheres grávidas e lactantes e pessoas idosas*” ^77^. Essa meta pode ser avaliada pelos indicadores coletados rotineiramente no Brasil e em estudos epidemiológicos em Portugal.

### Disseminação dos dados

Para além da realização de ações de vigilância alimentar e nutricional, é necessário que essas ações produzam efeitos na gestão das políticas públicas ^4^. Nesse sentido, a PNPS ^30^ do Brasil e o Plano de Ação Europeu para a Alimentação e Nutrição (*European Food and Nutrition Action Plan*) 2015-2020 ^78^ valorizam a utilização de múltiplas abordagens na geração e na análise de informações sobre as condições de saúde para subsidiar a tomada de decisão. Para isso, é necessário que as fontes de dados estejam devidamente organizadas e que os indicadores selecionados representem a situação nutricional da população. A linguagem utilizada nesses meios de disseminação é técnica, assim, os dois países ainda podem avançar na tradução do conhecimento técnico-científico para uma linguagem que influencie os tomadores de decisão. A disseminação dos resultados também pode ser realizada tendo como público-alvo grupos de pessoas, como organizações não governamentais e pesquisadores. A escolha do idioma pode contribuir para a disseminação; em alguns documentos de Portugal, por exemplo, observou-se que o sumário executivo era redigido em português e em inglês ^74^. Essa iniciativa poderia ser adotada nos documentos brasileiros com o intuito de disseminar as ações de vigilância alimentar e nutricional para indivíduos não usuários da língua portuguesa.

### Sustentabilidade

Ademais, importa refletir sobre a sustentabilidade das ações de vigilância alimentar e nutricional nos dois países. Em Portugal e no Brasil, os dois últimos inquéritos estão separados por 35 anos e 13 anos, respectivamente. Tal lacuna implicou a ausência de informações sobre o estado nutricional infantil durante sete gerações em Portugal e, virtualmente, três no Brasil ^55^ ^,^ ^69^ ^,^ ^79^. Nesses dois períodos, os dados disponíveis não foram suficientes para preencher a lacuna de informações causada pela ausência de inquéritos e, por extensão, houve impacto no processo de planejamento das políticas públicas. No Brasil, ainda há necessidade de mais informações sobre a confiabilidade dos indicadores para a avaliação da situação nutricional provenientes do SISVAN, as quais devem ser elucidadas futuramente. Em Portugal, o COSI se apresenta como uma estratégia para o monitoramento da obesidade em crianças do 1º ciclo do Ensino Básico a partir da abordagem de rede de escolas sentinelas ^22^.

A relevância do monitoramento do estado nutricional e do consumo alimentar em populações vem motivando a análise desse tema a partir de sistemas nacionais de vigilância alimentar e nutricional. Em revisão dos sistemas de vigilância alimentar e nutricional em países em desenvolvimento localizados na América, na África, na Ásia e no Oriente Médio, 31 sistemas nacionais de vigilância alimentar e nutricional foram identificados; desses, 16 continham informação completa e 15 informação parcial ^80^. Dois desafios foram observados na maioria dos sistemas: capacidade limitada de apropriação da vigilância pelos governos e incerteza quanto ao financiamento das ações ^81^. Em análise recente, Peters et al. ^81^ identificaram 82 programas de vigilância alimentar e nutricional em 18 países do Sudeste Asiático e do Pacífico Ocidental. A maioria dos programas de vigilância alimentar e nutricional foram implementados e eram coordenados pelos ministérios da saúde dos países ^81^. Todavia, muitos programas de vigilância foram implementados em bases pouco consistentes e dependiam de apoio financeiro de agências externas ^81^.

Brasil e Portugal compartilham fontes de dados semelhantes àquelas identificadas nos estudos apresentados no parágrafo anterior: inquéritos de base populacional e dados provenientes dos serviços públicos de saúde local. No Brasil e em Portugal, as políticas de saúde valorizam a vigilância alimentar e nutricional, porém, alguns aspectos podem ser aprimorados. Em Portugal, conforme mencionado, o inquérito IAN-AF 2015-2016 foi conduzido por um consórcio, incluindo na sua maioria instituições de Ensino Superior, e foi financiado por uma iniciativa pontual no âmbito do Espaço Econômico Europeu (EEA Grants, Iniciativas em Saúde Pública) ^55^. O Brasil adota o conceito ampliado de vigilância alimentar e nutricional com a utilização de diferentes fontes de dados. Nesse caso, a irregularidade do financiamento de inquéritos segue como o maior desafio ^82^.

Neste estudo, temos limitações que devem ser reportadas. Nós realizamos uma pesquisa ampla nos *websites* dos governos brasileiro e português; contudo, não podemos afirmar que todos os documentos relevantes para a descrição e a comparação das ações de vigilância alimentar e nutricional tenham sido recuperados. Os documentos são vestígios de uma época que tem os seus próprios contextos sociais e políticos, e podem não traduzir efetivamente as condições da realidade, sendo, por vezes, a expressão de uma vontade política não concretizada ou concretizada parcialmente.

## Conclusão

No Brasil, as ações de vigilância alimentar e nutricional foram fortalecidas em virtude de um conjunto de políticas do campo da Saúde Pública e da demanda gerada pela necessidade do acompanhamento das condicionalidades dos programas de transferência condicionada de renda. Em Portugal, a desarticulação das ações de vigilância alimentar e nutricional foi superada em parte com o programa nacional e com a EIPAS. Em ambos os países, as estratégias precisam ser aprimoradas em relação à regularidade do registro de dados, à harmonização dos indicadores de consumo alimentar (de modo a permitir a comparação entre países e no tempo) e à disseminação das informações de maneira organizada e em linguagem acessível para a utilização por gestores e pela sociedade civil.

A vigilância alimentar e nutricional baseada nos serviços de APS é uma das ações exitosas conduzidas no Brasil. Essa ação poderia complementar o atual modelo de vigilância alimentar e nutricional adotado em Portugal. Assim como no SUS do Brasil, os cuidados primários são priorizados no Sistema Nacional de Saúde (SNS) de Portugal. Assim, a implementação de um sistema nos moldes do SISVAN traria ganhos de sensibilidade ao monitoramento realizado em Portugal. A EIPAS é uma estratégia intersetorial conduzida por Portugal na perspectiva de Saúde em Todas as Políticas (STP) ^67^. Na EIPAS, foram propostas 51 medidas de intervenção para a promoção da alimentação saudável acordadas por sete ministérios ^67^. Os dados da vigilância alimentar e nutricional são utilizados no monitoramento das metas da EIPAS ^67^. Um exemplo de medida intersetorial foi a adoção de imposto para bebidas adicionadas de açúcar ^67^. Em contraponto, no Brasil, concentrados de refrigerantes produzidos na zona franca de Manaus recebem incentivos fiscais. A importância da intersetorialidade é recorrente nos documentos identificados para o Brasil. Nesses mesmos documentos, ressalta-se a necessidade de aprimorar a intersetorialidade. O modelo de implementação da EIPAS pode servir de subsídio para a formulação de políticas públicas de alimentação e nutrição no Brasil.
