# Supplementary material for: Food and nutrition surveillance actions in Brazil and Portugal: a comparative documentary analysis
Source: Cad Saude Publica. 2024 Sep 16;40(8):e00189823. [Article in Portuguese] doi: 10.1590/0102-311XPT189823 (PMC11405017; doi:10.1590/0102-311XPT189823)
Supplement: Supplementary file 1 [file 1678-4464-csp-40-08-PT189823-s.pdf]

Quadro S1 Documentos relacionados à vigilância alimentar e nutricional no Brasil, 1945 a 2022.

DOCUMENTO	TIPO	SÍNTESE
Decreto-lei nº 7.328, de 17 de fevereiro de 1945[1]	Decreto-lei	Cria, no Conselho Federal de Comércio Exterior, a Comissão Nacional de Alimentação. Atribuições da comissão: 1) estudar e propor as normas da política nacional de alimentação; 2) estudar o estado de nutrição e os hábitos alimentares da população brasileira, considerando o respectivo padrão de vida; 3) acompanhar e estimular as pesquisas relativas às questões e problemas de alimentação, propondo os auxílios que julgar necessários ou convenientes; 4) trabalhar pela correção de defeitos e deficiências da dieta brasileira, estimulando e acompanhando as devidas campanhas educativas; 5) concorrer para o desenvolvimento da indústria de desidratação dos alimentos no Brasil.
Lei nº 970, de 16 de dezembro de 1949[2]	Lei	Dispõe sobre as atribuições, organização e funcionamento do Conselho Nacional de Economia. Transfere para o Ministério da Educação e Saúde, a Comissão de Alimentação, criada pelo Decreto-lei nº 7.328, de 17 de fevereiro de 1945.
Lei nº 1.920, de 25 de julho de 1953[3]	Lei	Cria o Ministério da Saúde. Ao Ministério da Saúde são transferidos todos os órgãos e serviços do antigo Ministério da Educação e Saúde.
Lei nº 5.829, de 30 de novembro de 1972[4]	Lei	Cria o Instituto Nacional de Alimentação e Nutrição (INAN). Competências do INAN: 1) assistir o Governo na formulação da política nacional de alimentação e nutrição, 2) elaborar e propor o Programa Nacional de Alimentação e Nutrição (PRONAN), 3) funcionar como órgão central das atividades de alimentação e nutrição.
Decreto nº 72.034, de 30 de março de 1973[5]	Decreto	Institui o Programa Nacional de Alimentação e Nutrição (PRONAN), com a finalidade de acelerar a melhoria das condições de alimentação e nutrição da população, e consequentemente, contribuir para a elevação de seus padrões de saúde, índices de produtividade e níveis de renda. Aprova o I PRONAN com vigência 1973-1974.
Decreto nº 77.116, de 6 de fevereiro de 1976[6]	Decreto	O decreto estabelece diretrizes para a ação do Governo na área de Alimentação e Nutrição, aprova o Programa Nacional de Alimentação e Nutrição - PRONAN para o período de 1976 a 1979. Diretrizes gerais do II PRONAN: 1) racionalização da assistência e da educação na área da alimentação e da nutrição; 2) estímulo à produção, armazenagem, transporte e comercialização de alimentos básicos necessários aos programas oficiais de suplementação alimentar, mediante, especialmente, concessão de incentivos financeiros, fiscais e de mercado aos pequenos produtores cooperativados; 3) combate a carências nutricionais; 4) incentivo a estudos e pesquisas para melhoria dos padrões e das condições de alimentação e de nutrição; 5) apoio à capacitação de recursos humanos para os serviços técnicos referentes à alimentação e nutrição; 6) expansão do sistema de alimentação do trabalhador, através de concessão de financiamentos e de incentivos às empresas e instituições civis de empregados e empregadores; 7) organização de sistema integrado de aquisição, armazenamento e distribuição de alimentos destinados aos programas de suplementação alimentar.
Programa Nacional de Alimentação e Nutrição: PRONAN 1976/1979[7]	Documento técnico	O documento trata do PRONAN 1976-1979, que tinha como finalidade a redução do nível de pobreza absoluta e apoio ao pequeno produtor agrícola. O II PRONAN é um programa intersetorial com ações desenvolvidas em conjunto por diferentes ministérios. Diretrizes gerais de ação: 1) racionalização da oferta de serviços de suplementação alimentar, 2) racionalização do sistema de produção e comercialização de alimentos, 3) combate às carências nutricionais específicas, 4) estudos e pesquisas em alimentação e nutrição, 5) capacitação e aperfeiçoamento de recursos humanos. Programas e projetos do II PRONAN: 1) programa de suplementação alimentar, 2) programa de apoio ao pequeno produtor, 3) programa de atividades de apoio, 4) mecanismos de incentivos a programas de alimentação do trabalhador, com ênfase nos de baixa renda. O documento também trata do projeto de lei que dispõe sobre a dedução, do lucro tributável, para fins do imposto sobre a renda das pessoas jurídicas, o dobro das despesas realizadas em programas de alimentação do trabalhador.
Programa Nacional de Alimentação e Nutrição: abordagem dos aspectos multissetoriais de sua atuação[8]	Documento técnico	O documento foi apresentado na Conferência Internacional sobre Planejamento de Alimentação e Nutrição realizada na Guatemala. No documento são apresentados os projetos desenvolvidos no âmbito do PRONAN nas áreas prioritárias: 1) suplementação alimentar - programa de nutrição em saúde (PNS), projeto de abastecimento de alimentos básicos em áreas de baixa renda (PROAB), programa de nutrição escolar (PNE), programa de complementação alimentar (PCA), programa de alimentação do trabalhador (PAT); 2) racionalização do sistema de produção e comercialização de alimentos - projeto de produção de alimentos e nutrição em áreas rurais de baixa renda do nordeste, projeto de aquisição de alimentos básicos em áreas rurais de baixa renda (PROCAB); 3) atividades de complementação e apoio - estudos e pesquisas, testes experimentais de suplementação alimentar, desenvolvimento de recursos humanos, combate às carências nutricionais específicas, atividades de incentivo ao aleitamento materno.

Portaria nº 1.156, de 31 de agosto de 1990[9]	Portaria	1) Institui o Sistema de Vigilância Alimentar e Nutricional (SISVAN) no Ministério da Saúde. 2) Objetivos: manter o diagnóstico atualizado da situação do país, no que se refere aos problemas da área de alimentação e nutrição que possuem relevância em termos de saúde pública; identificar as áreas geográficas e grupos populacionais sob risco avaliando as tendências temporais de evolução dos problemas detectados; reunir dados que possibilitem identificar e ponderar os fatores mais relevantes na gênese desses problemas; oferecer subsídios ao planejamento e à execução de medidas para a melhoria da situação alimentar e nutricional da população brasileira.
Lei nº 8.080, de 19 de setembro de 1990[10]	Lei	1) Dispõe sobre as condições para a promoção, a proteção e a recuperação da saúde, a organização e o funcionamento dos serviços correspondentes e dá outras providências. 2) Inclui a vigilância nutricional e a orientação alimentar no campo de atuação do Sistema Único de Saúde (SUS), artigo 6º, item IV.
Portaria nº 80, de 16 de outubro de 1990[11]	Portaria	Constitui o Comitê Assessor do SISVAN, com a finalidade de oferecer apoio técnico-operacional à implementação do Sistema de Vigilância Alimentar e Nutricional.
Portaria nº 710, de 10 de junho de 1999[12]	Portaria	Aprova a Política Nacional de Alimentação e Nutrição (PNAN).
Política Nacional de Alimentação e Nutrição (2ª edição revista)[13]	Política	A PNAN de 1999 apresentava sete diretrizes programáticas e como fio norteador o Direito Humano à Alimentação e a Segurança Alimentar e Nutricional: 1) estímulo às ações intersetoriais com vistas ao acesso universal aos alimentos, 2) garantia da segurança e da qualidade dos alimentos e da prestação de serviços neste contexto, 3) monitoramento da situação alimentar e nutricional, 4) promoção de práticas alimentares e estilos de vida saudáveis, 5) prevenção e controle dos distúrbios nutricionais e das doenças associadas à alimentação e nutrição, 6) promoção de linhas de investigação, 7) desenvolvimento e capacitação de recursos humanos. Para o monitoramento da situação alimentar e nutricional apresentou a necessidade do fortalecimento e da ampliação do Sistema de Vigilância Alimentar e Nutricional. Além das diretrizes o documento apresenta as responsabilidades institucionais de cada nível de governo.
Programa de Combate às Carências Nutricionais – PCCN[14]	Relatório	O documento é um relatório do Programa de Combate às Carência Nutricionais, instituído em 23 de março de 1998, e descreve o conceito, funcionamento, requisitos e responsabilidades. O PCCN tinha como objetivo reduzir e controlar a desnutrição infantil em geral e as carências nutricionais específicas, principalmente a anemia ferropriva, a hipovitaminose A e incentivar a prática do aleitamento materno. Em relação ao funcionamento, preconizava-se o acompanhamento do estado nutricional das crianças no Sistema de Vigilância Alimentar e Nutricional. Destaca-se que o PCCN se seguiu ao Programa "Leite é Saúde" de modo a lidar com as dificuldades de implementação deste.
Medida Provisória nº 2.206-1, de 6 de setembro de 2001[15]	Medida Provisória	A medida provisória criou o Programa Nacional de Renda Mínima vinculado à saúde: "Bolsa Alimentação". O Programa tinha como público alvo gestantes, nutrizes e crianças de seis meses a seis anos e 11 meses de idade.
Decreto nº 3.934, de 20 de setembro de 2001[16]	Decreto	O Decreto aprova o Regulamento do Programa Nacional de Renda Mínima vinculado à saúde: "Bolsa Alimentação". As condicionalidades no campo da saúde descritas no decreto são ações básicas de saúde como pré-natal, vacinação, acompanhamento do crescimento e desenvolvimento, incentivo ao aleitamento materno e atividades educativas em saúde. O "Bolsa Alimentação" substituiu o Incentivo ao Combate às Carências Nutricionais.
Portaria nº 2.246, de 18 de outubro de 2004[17]	Portaria	1) Institui e divulga orientações básicas para a implementação das Ações de Vigilância Alimentar e Nutricional, no âmbito das ações básicas de saúde do SUS em todo o território nacional. 2) Aprovar o Manual de Orientações Básicas para a Coleta, Processamento, Análise de Dados e Informação em Serviços de Saúde para o SISVAN.
Vigilância alimentar e nutricional – Sisvan: orientações básicas para a coleta, processamento, análise de dados e informação em serviços de saúde[18]	Manual técnico	1) Fornecer aos profissionais responsáveis pelas ações de vigilância alimentar e nutricional subsídios para a prática. 2) O manual é dividido em duas partes, na primeira são abordados objetivo, definição e histórico da vigilância alimentar e nutricional e na segunda são abordados aspectos relativos à avaliação do estado nutricional.
Lei nº 10.836, de 9 de janeiro de 2004[19]	Lei	A Lei cria o Programa Bolsa Família (PBF) que é destinado às ações de transferência de renda com condicionalidades. O PBF visa a unificação de diferentes programas entre eles o Programa Nacional de Renda Mínima vinculada à Saúde - Bolsa Alimentação. A concessão dos benefícios depende do cumprimento de condicionalidades tais como pré-natal, acompanhamento nutricional, acompanhamento de saúde, frequência escolar mínima de 85%. Nesta lei também é criado o Conselho Gestor Interministerial do PBF.
Decreto nº 5.209, de 17 de setembro de 2004[20]	Decreto	O decreto regulamenta a Lei nº 10.836 que criou o PBF. Objetivos básicos do PBF: I) promover o acesso à rede de serviços públicos, em especial, de saúde, educação e assistência social; II) combater a fome e promover a segurança alimentar e

		nutricional; III) estimular a emancipação sustentada das famílias que vivem em situação de pobreza e extrema pobreza; IV) combater a pobreza; e V) promover a intersetorialidade, a complementariedade e a sinergia das ações sociais do Poder Público. O Ministério da Saúde é responsável pelo acompanhamento do crescimento e desenvolvimento infantil, da assistência ao pré-natal e ao puerpério, da vacinação, bem como da vigilância alimentar e nutricional de crianças menores de 7 anos.
Portaria Interministerial nº 2.509, de 18 de novembro de 2004[21]	Portaria	Dispõe sobre as atribuições e normas para a oferta e o monitoramento das ações de saúde relativas às condicionalidades das famílias beneficiárias do PBF. Em relação a vigilância alimentar e nutricional, realizar o acompanhamento das crianças menores de 7 anos.
Portaria nº 2.608/GM, de 28 de dezembro de 2005[22]	Portaria	A portaria define recursos financeiros do Teto Financeiro de Vigilância em Saúde, para incentivar a estruturação de ações de Vigilância e Prevenção de Doenças e Agravos Não-Transmissíveis por parte das Secretarias Estaduais e Secretarias Municipais de Saúde das capitais. No Art. 2º, item IV: implementação da vigilância de saúde por meio de instrumentos de monitoramento, prevenção e vigilância de morbimortalidade e dos fatores de risco relativos às doenças e agravos não-transmissíveis, utilizando os sistemas de informação existentes na análise da situação de saúde e no planejamento das ações de promoção da saúde e prevenção das doenças e agravos não-transmissíveis.
Portaria nº 648/GM, de 28 de março de 2006[23]	Portaria	Aprova a Política Nacional de Atenção Básica (PNAB), estabelecendo a revisão de diretrizes e normas para a organização da Atenção Básica para o Programa Saúde da Família (PSF) e o Programa Agentes Comunitários de Saúde (PACS).
Política Nacional de Atenção Básica[24]	Política	A Política estabelece a suspensão do repasse de recursos aos municípios e ao Distrito Federal quando não houver alimentação regular dos bancos de dados nacionais de informação, entre os quais o SISVAN.
Portaria nº 687, de 30 de março de 2006[25]	Portaria	Aprova a Política Nacional de Promoção da Saúde (PNPS).
Política Nacional de Promoção da Saúde (3ª edição)[26]	Política	A PNPS discute a importância da vigilância em saúde para a promoção à saúde e apresenta como objetivo a implementação de ações de vigilância alimentar e nutricional para a prevenção e controle dos agravos e doenças decorrentes da má alimentação. Em relação a reorientação dos serviços de saúde com ênfase na atenção básica coloca a necessidade de incorporação do componente alimentar no SISVAN de forma a permitir o diagnóstico e o desenvolvimento de ações para a promoção da alimentação saudável, reforço da implantação do SISVAN como instrumento de avaliação e de subsídio para o planejamento de ações que promovam a segurança alimentar e nutricional em nível local.
Portaria Interministerial nº 1.010, de 08 de maio de 2006[27]	Portaria	A portaria institui as diretrizes para a Promoção da Alimentação Saudável nas escolas de educação infantil, fundamental e nível médio das redes públicas e privadas em âmbito nacional. No que concerne a vigilância alimentar e nutricional, inclui como eixo prioritário o monitoramento da situação nutricional dos escolares.
Portaria nº 1.097, de 22 de maio de 2006[28]	Portaria	A portaria define que o processo de Programação Pactuada e Integrada da Assistência em Saúde seja um processo instituído no âmbito do SUS. A Programação Pactuada e Integrada da Assistência em Saúde tem por objetivo organizar a rede de serviços e definir os limites financeiros destinados à assistência da população própria e das referências recebidas de outros municípios.
Decreto nº 6.286, de 5 de dezembro de 2007[29]	Decreto	O decreto institui o Programa Saúde na Escola (PSE) com a finalidade de contribuir para a formação integral dos estudantes da rede pública de educação básica por meio de ações de prevenção, promoção e atenção à saúde.
Manual de orientações sobre o Bolsa Família na Saúde – 2ª edição[30]	Manual técnico	O documento apresenta: 1) informações sobre o PBF (o que é, quando foi criado, objetivo, público alvo, os benefícios, as condicionalidades); 2) o papel das Secretarias Municipais de Saúde; 3) a oferta das ações de saúde às famílias beneficiárias; 4) relação entre o SISVAN e o PBF; 5) orientação de digitação dos dados do mapa de acompanhamento do SISVAN.
Protocolos do Sistema de Vigilância Alimentar e Nutricional – SISVAN na assistência à saúde[31]	Protocolo	O documento tem por objetivo apresentar os protocolos do SISVAN por ciclo da vida. Para cada ciclo apresenta classificação do estado nutricional, principais agravos nutricionais e como diagnosticá-los, procedimentos para avaliação do perfil alimentar e nutricional, análise dos dados e orientações nutricionais.
Manual de orientações sobre o Bolsa Família na Saúde - 3ª edição[32]	Manual técnico	O documento é uma edição atualizada do manual lançado em 2007.
Matriz de ações de alimentação e nutrição na atenção básica de saúde[33]	Manual técnico	O objetivo do documento foi sistematizar e organizar as ações de alimentação e nutrição e do cuidado nutricional para fazerem parte do rol de ações de saúde da atenção básica, tendo como base a Política Nacional de Alimentação e Nutrição (PNAN). Pressupostos conceituais e organizacionais da matriz: a) sujeitos das ações - o indivíduo, a família e a comunidade; b) níveis de intervenção - gestão das ações de alimentação e nutrição e cuidado nutricional (diagnóstico, promoção da saúde,

		prevenção de doenças, tratamento/cuidado/assistência); c) caráter das ações - universais (aplicáveis a quaisquer fases da vida) e específicas (aplicáveis a determinadas fases da vida). Referente a gestão das ações, a matriz estabelece a importância da utilização do SISVAN para o planejamento das ações, e a necessidade de seu fortalecimento.
Portaria nº 3.642, de 11 de novembro de 2010[34]	Portaria	A portaria estabelece os critérios para alimentação dos Bancos de Dados Nacionais dos Sistemas de Informação da Atenção à Saúde. Definição da obrigatoriedade de alimentação mensal e sistemática dos Bancos de Dados Nacionais.
Portaria nº 4.279, de 30 de dezembro de 2010[35]	Portaria	A portaria estabelece as diretrizes para a organização da Rede de Atenção à Saúde, no âmbito do SUS, como estratégia para superar a fragmentação da atenção e da gestão das regiões de saúde e aperfeiçoar o funcionamento político institucional do SUS. A portaria indica como estratégia o fortalecimento das ações de âmbito coletivo da vigilância em saúde com as da assistência tanto coletiva quanto individual. E indica como estratégia a utilização dos sistemas de informação para a construção do diagnóstico da situação de saúde, a fim de produzir intervenções baseadas nas necessidades das populações.
Portaria nº 2.488, de 21 de outubro de 2011[36]	Portaria	Aprova a Política Nacional de Atenção Básica (PNAB), estabelecendo a revisão de diretrizes e normas para a organização da Atenção Básica, para a Estratégia Saúde da Família (ESF) e o Programa de Agentes Comunitários de Saúde (PACS).
Política Nacional de Atenção Básica[37]	Política	A Política reforça a importância da vigilância à saúde como um dos aspectos para coordenar a integralidade do cuidado. E estabelece como responsabilidade o desenvolvimento, implementação e disponibilização dos sistemas de informação a todas as esferas de governo.
Portaria nº 2.715, de 17 de novembro de 2011[38]	Portaria	Atualiza e aprova a PNAN, determina que os órgãos e entidades do Ministério da Saúde, cujas ações se relacionem com o tema objeto da Política ora aprovada, promovam a elaboração ou a readequação de seus planos, programas, projetos e atividades das diretrizes e responsabilidades nela estabelecidas.
Política Nacional de Alimentação e Nutrição[39]	Política	A PNAN tem como propósito a melhoria das condições de alimentação, nutrição e saúde da população brasileira, e apresenta nove diretrizes: 1) organização da atenção nutricional, 2) promoção da alimentação adequada e saudável, 3) vigilância alimentar e nutricional, 4) gestão das ações de alimentação e nutrição, 5) participação e controle social, 6) qualificação da força de trabalho, 7) controle e regulação dos alimentos, 8) pesquisa, inovação e conhecimento em alimentação e nutrição, 9) cooperação e articulação para a Segurança Alimentar e Nutricional. A PNAN destaca a importância do fortalecimento institucional da vigilância alimentar e nutricional para o planejamento da atenção nutricional e o desenvolvimento das ações.
Orientações para a coleta e análise de dados antropométricos em serviços de saúde: Norma Técnica do Sistema de Vigilância Alimentar e Nutricional (SISVAN)[40]	Norma técnica	O documento é dividido em três partes: 1) definições e conceitos para o diagnóstico e o acompanhamento do estado nutricional, 2) parâmetros preconizados pela vigilância nutricional para cada fase do curso da vida, 3) equipamentos antropométricos e a técnica de aferição.
Decreto nº 7.508, de 28 de junho de 2011[41]	Decreto	O decreto regulamenta a Lei nº 8.080, de 19 de setembro de 1990, para dispor sobre a organização do SUS, o planejamento da saúde, a assistência à saúde e a articulação interfederativa.
Portaria nº 2.975, de 14 de dezembro de 2011[42]	Portaria	Estabelece apoio financeiro aos Municípios e ao Distrito Federal na estruturação da Vigilância Alimentar e Nutricional para um diagnóstico nutricional e alimentar adequado e humanizado, por meio do provimento de equipamentos adequados para esse fim.
Manual orientador para aquisição de equipamentos antropométricos [Portaria nº 2.975, de 14 de dezembro de 2011][43]	Manual técnico	O documento apresenta as especificações para aquisição dos equipamentos antropométricos, na medida em que a aquisição e manutenção dos mesmos são fundamentais para a vigilância alimentar e nutricional, assim como o local onde estão dispostos.
Portaria nº 2.387, de 18 de outubro de 2012[44]	Portaria	A portaria instituiu a Agenda para Intensificação da Atenção Nutricional à Desnutrição Infantil (ANDI) em municípios com maior prevalência de déficit ponderal em crianças menores de 5 anos. A portaria considerou como desnutrição infantil moderada baixo peso para idade e como desnutrição infantil grave muito baixo peso para idade. Entre os fundamentos da Agenda destaca-se a vigilância alimentar e nutricional da população infantil. E entre os compromissos mínimos estipulados ao município destaca-se o acompanhamento da situação alimentar e nutricional da população por meio do SISVAN. As cinco metas da Agenda: I) aumentar o acompanhamento do estado nutricional de crianças menores de 5 (cinco)

		anos no SISVAN; II) investigar os casos de desnutrição e atraso no desenvolvimento infantil; III) aumentar o acompanhamento das condicionalidades de saúde das famílias beneficiárias do Programa Bolsa Família; IV) implementar ações de incentivo ao aleitamento materno e de promoção da alimentação complementar saudável para crianças de até 2 (dois) anos de idade em todas as unidades básicas de saúde do Município; e V) garantir a suplementação de ferro e de vitamina A para todas as crianças diagnosticadas com desnutrição.
Manual instrutivo para implementação da Agenda para Intensificação da Atenção Nutricional Infantil: Portaria nº 2.387, de 18 de outubro de 2012[45]	Manual	A Agenda para Intensificação da Atenção Nutricional à Desnutrição Infantil (ANDI) tinha como objetivo impulsionar o enfrentamento da desnutrição nos municípios com maior prevalência desse agravo em crianças menores de 5 anos, por meio da estruturação e da qualificação de ações de atenção à saúde da criança e da organização da atenção nutricional na Rede de Atenção à Saúde, no âmbito da atenção básica. A vigilância alimentar e nutricional tem papel fundamental na organização da atenção nutricional ao permitir o levantamento das condições do território e realizar a identificação das crianças com desnutrição. O manual instrutivo aborda os seguintes temas: vigilância alimentar e nutricional, acompanhamento das condicionalidades do PBF, investigação dos casos de desnutrição e atraso do desenvolvimento infantil, organização do cuidado à criança desnutrida (elaboração do Projeto Terapêutico Singular e do Projeto de Saúde do Território), promoção da alimentação saudável, prevenção de carências de micronutrientes e planejamento municipal para implementação da ANDI.
Portaria nº 2.446, de 11 de novembro de 2014[46]	Portaria	A Portaria redefine a Política Nacional de Promoção da Saúde (PNPS). A Política tem por objetivo geral promover a equidade e a melhoria das condições e modos de viver, ampliando a potencialidade da saúde individual e da saúde coletiva, reduzindo vulnerabilidades e riscos à saúde decorrentes dos determinantes sociais, econômicos, políticos, culturais e ambientais. A Política apresenta nove eixos operacionais, entre eles destaca-se o eixo vigilância, monitoramento e avaliação que trata da utilização de múltiplas abordagens na geração e na análise de informações sobre as condições de saúde para subsidiar a tomada de decisão. A Política apresenta oito temas prioritários, destacando-se a promoção de ações relativas à alimentação adequada e saudável.
Marco de referência da vigilância alimentar e nutricional na atenção básica[47]	Documento diretivo	O documento inicialmente apresenta o histórico da vigilância alimentar e nutricional e estabelece um paralelo com a vigilância em saúde. Em seguida, explana como a vigilância alimentar e nutricional está inserida no ciclo de gestão e produção do cuidado nos âmbitos individual e coletivo. Por fim, apresenta situações de aplicação da vigilância alimentar e nutricional na prática.
Orientações para avaliação de marcadores de consumo alimentar na atenção básica[48]	Manual orientador	O documento é dividido em quatro partes: 1) importância do acompanhamento da situação alimentar e nutricional, 2) orientações para a vigilância alimentar na atenção básica, 3) interpretação dos indicadores do módulo de consumo alimentar, 4) registro dos marcadores de consumo alimentar em sistemas de informação em saúde.
Portaria nº 2.436, de 21 de setembro de 2017[49]	Portaria	A portaria aprova a PNAB com vistas à revisão da regulamentação de implantação e operacionalização vigentes no âmbito do SUS, estabelecendo-se as diretrizes para a organização do componente Atenção Básica na Rede de Atenção à Saúde (RAS). A Política reforça a importância da integração do trabalho entre Atenção Básica e Vigilância em Saúde. Em relação aos sistemas de informação, a Política estabelece como responsabilidade de desenvolver, disponibilizar e implantar a todas as esferas de governo.
Manual operacional para uso do sistema de vigilância alimentar e nutricional [SISVAN – Versão 3.0][50]	Manual operacional [versão preliminar]	O documento apresenta orientações de uso do SISVAN web: aspectos gerais, bloco de cadastro, bloco de registro de acompanhamento, bloco de vinculações, bloco de relatórios, integração do SISVAN com outros sistemas, suporte ao sistema, materiais de apoio.
Política Nacional de Promoção da Saúde[51]	Política	Anexo I da Portaria de Consolidação nº 2, de 28 de setembro de 2017, que consolida as normas sobre as políticas nacionais de saúde do SUS. A Política Nacional de Promoção da Saúde tem como objetivo geral promover a equidade e a melhoria das condições e dos modos de viver, ampliando a potencialidade da saúde individual e coletiva e reduzindo vulnerabilidades e riscos à saúde decorrentes dos determinantes sociais, econômicos, políticos, culturais e ambientais. A Política apresenta nove eixos operacionais, entre eles destaca-se o eixo vigilância, monitoramento e avaliação que trata da utilização de múltiplas abordagens na geração e na análise de informações sobre as condições de saúde para subsidiar a tomada de decisão. A Política apresenta oito temas prioritários, destacando-se a promoção de ações relativas à alimentação adequada e saudável.
Resolução nº 588, de 12 de julho de 2018[52]	Resolução	A Resolução institui a Política Nacional de Vigilância em Saúde (PNVS). A PNVS tem como finalidade definir os princípios, as diretrizes e as estratégias a serem observadas pelas três esferas de gestão do SUS, para o desenvolvimento da vigilância em saúde.

Guia Política Nacional de Atenção Básica – Módulo 1: Integração Atenção Básica e Vigilância em Saúde[53]	Manual técnico	O guia tem como objetivo orientar gestores e profissionais de saúde para a realização da integração da atenção básica com a vigilância em saúde. O guia apresenta estratégias de integração relacionadas à organização no território e ao processo de trabalho das equipes. O processo de territorialização é um meio operacional para o desenvolvimento do vínculo entre os serviços de saúde e a população. O Guia orienta que as ações de vigilância em saúde devem ser transversais a todas as ações da equipe de atenção básica.
Portaria nº 1.862, de 10 de agosto de 2021[54]	Portaria	A Portaria institui a Estratégia Nacional para Prevenção e Atenção à Obesidade Infantil (Proteja). A Proteja tem como objetivo geral deter o avanço da obesidade infantil e contribuir para a melhoria da saúde e da nutrição das crianças. Entre os eixos de ação para implementação da Proteja destaca-se a vigilância alimentar e nutricional, promoção da saúde e de prevenção do ganho excessivo de peso, diagnóstico precoce e cuidado adequado às crianças, adolescentes e gestantes no âmbito da Atenção Primária à Saúde. Os indicadores para monitoramento no Proteja são: I) número de crianças com estado nutricional (peso e altura) avaliado; II) número de crianças com práticas alimentares (marcadores de consumo alimentar) avaliadas; III) número de atendimentos individuais para problema ou condição avaliada como obesidade em crianças; IV) prevalência de crianças com obesidade.
PROTEJA: Estratégia Nacional para Prevenção e Atenção à Obesidade Infantil: orientações técnicas [versão preliminar][55]	Manual orientador	O documento tem como objetivo apresentar a Estratégia Nacional para Prevenção e Atenção à Obesidade Infantil (Proteja) e apoiar a implementação de ações em nível municipal. O documento estabelece as seguintes estratégias para prevenção e atenção à obesidade infantil na Atenção Primária à Saúde: capacitação de profissionais de saúde, vigilância alimentar e nutricional, promoção do aleitamento materno e da alimentação adequada e saudável, promoção da atividade física e organização do cuidado às crianças com sobrepeso e obesidade. A vigilância alimentar e nutricional é fundamental para o diagnóstico precoce do excesso de peso e obesidade, e para identificar fatores de risco relacionados ao ganho excessivo de peso.
Instrutivo para o cuidado da criança e do adolescente com sobrepeso e obesidade no âmbito da Atenção Primária à Saúde [versão preliminar][56]	Manual	O Instrutivo é destinado aos profissionais da saúde para a oferta do cuidado a crianças e adolescentes diagnosticados com sobrepeso e obesidade. De acordo com o Instrutivo, as ações são iniciadas com a realização da avaliação contínua e sistemática do perfil nutricional de crianças e adolescentes, além de garantir a atenção integral às crianças com sobrepeso e obesidade. No mapa de determinantes, a vigilância alimentar e nutricional está disposta no ambiente medial/mesossistema. A vigilância alimentar e nutricional de crianças e adolescentes pode ocorrer em diferentes oportunidades: rotina dos serviços de saúde, buscas das famílias por atendimento, ações coordenadas nas escolas, campanhas no território, entre outras.
Lei nº 14.284, de 29 de dezembro de 2021[57]	Lei	A Lei institui o Programa Auxílio Brasil e o Programa Alimenta Brasil em substituição ao Programa Bolsa Família e ao Programa de Aquisição de Alimentos, respectivamente. Objetivos do Programa Auxílio Brasil: I) promover a cidadania com garantia de renda e apoiar, por meio dos benefícios e serviços ofertados pelo Suas, a articulação de políticas direcionadas aos beneficiários, com vistas à superação das vulnerabilidades sociais das famílias; II) reduzir as situações de pobreza e de extrema pobreza das famílias beneficiárias; III) promover, prioritariamente, o desenvolvimento das crianças e dos adolescentes, por meio de apoio financeiro a gestantes, a nutrizes, a crianças e a adolescentes em situação de pobreza ou de extrema pobreza; IV) promover o desenvolvimento das crianças na primeira infância, com foco na saúde e nos estímulos às habilidades física, cognitivas, linguísticas e socioafetivas; V) ampliar a oferta do atendimento das crianças em creches; VI) estimular crianças, adolescentes e jovens a terem desempenho científico e tecnológico de excelência; e VII) estimular e emancipação das famílias em situação de pobreza e de extrema pobreza, principalmente por meio: a) da inserção dos adolescentes maiores de 16 (dezesseis) anos, dos jovens e dos adultos no mercado de trabalho; b) da integração das políticas socioassistenciais com as políticas de promoção à inclusão produtiva; e c) do incentivo ao empreendedorismo, ao microcrédito e à inserção no mercado de trabalho formal. A manutenção da condição de família beneficiária dependerá do cumprimento das seguintes condicionalidades: I) a realização de pré-natal; II) cumprimento do calendário nacional de vacinação e do acompanhamento do estado nutricional; e III) frequência escolar mínima.
Matriz para Organização dos Cuidados em Alimentação e Nutrição na Atenção Primária à Saúde [versão preliminar][58]	Manual técnico	O documento apresenta proposta para organização das ações essenciais em Alimentação e Nutrição na Atenção Primária à Saúde (APS). Atributos essenciais da APS: atenção ao primeiro contato (acesso), longitudinalidade, integralidade e coordenação do cuidado. Atributos derivados da APS: centralidade na família, orientação comunitária e competência cultural. A atenção nutricional pode contribuir para a realização dos atributos da APS. A vigilância alimentar e nutricional é uma das ações/cuidados que compõem a atenção nutricional no SUS, com o objetivo de realizar o acompanhamento da situação alimentar e nutricional dos indivíduos e coletividades. No documento são apresentados os equipamentos necessários e a estrutura adequada para a realização da atenção nutricional na APS. A abordagem de práticas de cuidado em Alimentação e Nutrição proposta no documento é a do

		cuidado emancipador, e são considerados como sujeitos do cuidado: os indivíduos, as famílias e as comunidades.
Guia para a Organização da Vigilância Alimentar e Nutricional na Atenção Primária à Saúde[59]	Manual técnico	O Guia tem como objetivo orientar profissionais e gestores de saúde na realização das ações de vigilância alimentar e nutricional. O material é dividido em apresentação, estruturação da vigilância alimentar e nutricional (recursos físicos e materiais, humanos e financeiros), realização da vigilância alimentar e nutricional (avaliação antropométrica do estado nutricional e consumo alimentar por meio dos marcadores) e o exercício da atitude de vigilância a partir do diagnóstico observado. Neste documento, é destacada a importância da vigilância alimentar e nutricional para organização de estratégias, programas e linhas de cuidado.

Referências

1. Brasil. Presidência da República. Decreto-lei no 7.328, de 17 de fevereiro de 1945 [Internet]. 7328 fev 17, 1945. Disponível em: http://www.planalto.gov.br/ccivil_03/Decreto-Lei/1937-1946/Del7328.htm

2. Brasil. Presidência da República. Lei no 970, de 16 de dezembro de 1949 [Internet]. 970 dez 16, 1949. Disponível em: http://www.planalto.gov.br/ccivil_03/leis/1930-1949/l0970.htm

3. Brasil. Presidência da República. Lei no 1.920, de 25 de julho de 1953 [Internet]. 1920 jul 25, 1953. Disponível em: http://www.planalto.gov.br/ccivil_03/leis/1950-1969/l1920.htm#:~:text=LEI%20No%201.920%2C%20DE%2025%20DE%20JULHO%20DE%201953.&text=Cria%20o%20Minist%C3%A9rio%20da%20Sa%C3%BAde,problemas%20atinentes%20%C3%A0%20sa%C3%BAde%20humana.

4. Brasil. Presidência da República. Lei no 5.829, de 30 de novembro de 1972 [Internet]. 5829 nov 30, 1972. Disponível em: https://www2.camara.leg.br/legin/fed/lei/1970-1979/lei-5829-30-novembro-1972-358082-publicacaooriginal-1-pl.html

5. Brasil. Presidência da República. Decreto no 72.034, de 30 de março de 1973 [Internet]. mar 30, 1973. Disponível em: https://www2.camara.leg.br/legin/fed/decret/1970-1979/decreto-72034-30-marco-1973-420497-publicacaooriginal-1-pe.html

6. Brasil. Presidência da República. Decreto no 77.116, de 6 de fevereiro de 1976 [Internet]. fev 6, 1976. Disponível em: https://www2.camara.leg.br/legin/fed/decret/1970-1979/decreto-77116-6-fevereiro-1976-425734-publicacaooriginal-1-pe.html#:~:text=Estabelece%20diretrizes%20para%20a%20a%C3%A7%C3%A3o,PRONAN%20e%20d%C3%A1%20outras%20provid%C3%AAncias.

7. Brasil. Instituto Nacional de Alimentação e Nutrição (INAN). Programa Nacional de Alimentação e Nutrição: PRONAN 1976/1979 [Internet]. Ministério da Saúde; 1976 [citado 4 de setembro de 2022]. Disponível em: https://bvsms.saude.gov.br/bvs/publicacoes/programa_nacional_alimentacao_nutricao_1976_1979_documento_tecnico.pdf

8. Brasil. Ministério da Saúde, Instituto Nacional de Alimentação e Nutrição (INAN). Programa Nacional de Alimentação e Nutrição: abordagem dos aspectos multissetoriais de sua atuação [Internet]. Ministério da Saúde; 1980 [citado 4 de setembro de 2022]. Disponível em: https://bvsms.saude.gov.br/bvs/publicacoes/programa_nacional_alimentacao_nutricao_abordagem.pdf

9. Brasil. Ministério da Saúde. Portaria no 1.156, de 31 de agosto de 1990 [Internet]. Disponível em: http://189.28.128.100/nutricao/docs/legislacao/portaria_sisvan.pdf

10. Brasil. Presidência da República. Lei no 8.080, de 19 de setembro de 1990 [Internet]. Disponível em: http://www.planalto.gov.br/ccivil_03/leis/l8080.htm

11. Brasil. Ministério da Saúde. Portaria no 80, de 16 de outubro de 1990 [Internet]. 80 out 16, 1990. Disponível em: http://189.28.128.100/nutricao/docs/legislacao/portaria_sisvan.pdf

12. Brasil. Ministério da Saúde. Portaria no 710, de 10 de junho de 1999 [Internet]. 710 jun 10, 1999. Disponível em: https://bvsms.saude.gov.br/bvs/saudelegis/gm/1999/prt0710_10_06_1999.html

13. Brasil. Ministério da Saúde. Secretaria de Atenção à Saúde. Departamento de Atenção Básica. Política Nacional de Alimentação e Nutrição (a) [Internet]. Ministério da Saúde; 2008 [citado 4 de setembro de 2022]. Disponível em: https://bvsms.saude.gov.br/bvs/publicacoes/politica_nacional_alimentacao_nutricao_2ed.pdf

14. Brasil. Ministério da Saúde. Programa de Combate às Carências Nutricionais - PCCN [Internet]. Brasília: Ministério da Saúde; 2001 jan [citado 4 de setembro de 2022] p. 36. Disponível em: https://bvsms.saude.gov.br/bvs/publicacoes/cd10_13.pdf

15. Brasil. Presidência da República. Medida Provisória no 2.206-1, de 6 de setembro de 2001 [Internet]. 2206 set 6, 2001. Disponível em: http://www.planalto.gov.br/ccivil_03/mpv/antigas_2001/2206-1.htm

16. Brasil. Presidência da República. Decreto no 3.934, de 20 de setembro de 2001 [Internet]. 3934 set 20, 2001. Disponível em: http://www.planalto.gov.br/ccivil_03/decreto/2001/D3934htm.htm

17. Brasil. Ministério da Saúde. Portaria no 2.246, de 18 de outubro de 2004 [Internet]. 2246 out 18, 2004. Disponível em: https://bvsms.saude.gov.br/bvs/saudelegis/gm/2004/prt2246_18_10_2004.html

18. Brasil. Ministério da Saúde. Vigilância alimentar e nutricional - Sisvan: orientações básicas para a coleta, processamento, análise de dados e informação em serviços de saúde [Internet]. Brasília; 2004 [citado 4 de setembro de 2022]. Disponível em: http://189.28.128.100/nutricao/docs/geral/orientacoes_basicas_sisvan.pdf

19. Brasil. Ministério da Saúde. Lei no 10.836, de 9 de janeiro de 2004 [Internet]. 10836 jan 9, 2004. Disponível em: https://www2.camara.leg.br/legin/fed/lei/2004/lei-10836-9-janeiro-2004-490604-publicacaooriginal-1-pl.html

20. Brasil. Presidência da República. Decreto no 5.209, de 17 de setembro de 2004 [Internet]. Disponível em: http://www.mds.gov.br/webarquivos/legislacao/bolsa_familia/decreto/decreto_no_5209_de_17.09.2004-1.pdf

21. Brasil. Ministério da Saúde. Ministério do Desenvolvimento Social. Portaria Interministerial no 2.509, de 18 de novembro de 2004 [Internet]. 2509 nov 18, 2004. Disponível em: https://bvsms.saude.gov.br/bvs/publicacoes/manual_orientacoes_bolsa_familia_2ed.pdf

22. Brasil. Ministério da Saúde. Portaria no 2.608/GM, de 28 de dezembro de 2005 [Internet]. 2608 dez 28, 2005. Disponível em: http://189.28.128.100/nutricao/docs/legislacao/portaria2608_28_12_05.pdf

23. Brasil. Ministério da Saúde. Portaria no 648/GM, de 28 de março de 2006 [Internet]. 648 mar 28, 2006. Disponível em: https://bvsms.saude.gov.br/bvs/publicacoes/prtGM648_20060328.pdf

24. Brasil. Ministério da Saúde. Secretaria de Atenção à Saúde. Departamento de Atenção Básica. Política Nacional de Atenção Básica (a) [Internet]. Ministério da Saúde; 2006 [citado 4 de setembro de 2022]. Disponível em: https://bvsms.saude.gov.br/bvs/publicacoes/politica_nacional_atencao_basica_2006.pdf

25. Brasil. Ministério da Saúde. Portaria no 687, de 30 de março de 2006 [Internet]. 687 mar 30, 2006. Disponível em: http://189.28.128.100/dab/docs/legislacao/portaria687_30_03_06.pdf

26. Brasil. Ministério da Saúde. Secretaria de Vigilância em Saúde. Secretaria de Atenção à Saúde. Política Nacional de Promoção da Saúde (a) [Internet]. Ministério da Saúde; 2010 [citado 4 de setembro de 2022]. Disponível em: https://bvsms.saude.gov.br/bvs/publicacoes/politica_nacional_promocao_saude_3ed.pdf

27. Brasil. Ministério da Saúde. Ministério da Educação. Portaria Interministerial no 1.010, de 08 de maio de 2006 [Internet]. 1010 maio 8, 2006. Disponível em: https://bvsms.saude.gov.br/bvs/saudelegis/gm/2006/pri1010_08_05_2006.html

28. Brasil. Ministério da Saúde. Portaria no 1.097, de 22 de maio de 2006 [Internet]. 1097 maio 22, 2006. Disponível em: https://bvsms.saude.gov.br/bvs/saudelegis/gm/2006/prt1097_22_05_2006_comp.html

29. Brasil. Presidência da República. Decreto no 6.286, de 5 de dezembro de 2007 [Internet]. 6286 dez 5, 2007. Disponível em: http://www.planalto.gov.br/ccivil_03/_ato2007-2010/2007/decreto/d6286.htm

30. Brasil. Ministério da Saúde. Secretaria de Atenção à Saúde. Departamento de Atenção Básica. Manual de orientações sobre o Bolsa Família na Saúde - 2a edição [Internet]. Ministério da Saúde; 2007 [citado 4 de setembro de 2022]. Disponível em: https://bvsms.saude.gov.br/bvs/publicacoes/manual_orientacoes_bolsa_familia_2ed.pdf

31. Brasil. Ministério da Saúde. Secretaria de Atenção à Saúde. Departamento de Atenção Básica. Protocolos do Sistema de Vigilância Alimentar e Nutricional - SISVAN na assistência à saúde [Internet]. Brasília: Ministério da Saúde; 2008 [citado 21 de fevereiro de 2022]. Disponível em: https://bvsms.saude.gov.br/bvs/publicacoes/protocolo_sistema_vigilancia_alimnetar.pdf

32. Brasil. Ministério da Saúde. Secretaria de Atenção à Saúde. Departamento de Atenção Básica. Manual de orientações sobre o Bolsa Família na Saúde - 3a edição [Internet]. Ministério da Saúde; 2010 [citado 4 de setembro de 2022]. Disponível em: https://bvsms.saude.gov.br/bvs/publicacoes/manual_orientacao_sobre_bolsa_familia.PDF

33. Brasil. Ministério da Saúde. Secretaria de Atenção à Saúde. Departamento de Atenção Básica. Matriz de ações de alimentação e nutrição na atenção básica de saúde. [Internet]. Brasília: Ministério da Saúde; 2009 [citado 11 de setembro de 2022] p. 78. (Série A. Normas e Manuais Técnicos). Disponível em: https://bvsms.saude.gov.br/bvs/publicacoes/matriz_alimentacao_nutricao.pdf

34. Brasil. Ministério da Saúde. Portaria no 3.642, de 11 de novembro de 2010 [Internet]. 3642 nov 11, 2010. Disponível em: https://bvsms.saude.gov.br/bvs/saudelegis/gm/2010/prt3462_11_11_2010_comp.html

35. Brasil. Ministério da Saúde. Portaria no 4.279, de 30 de dezembro de 2010 [Internet]. 4279 dez 30, 2010. Disponível em: https://bvsms.saude.gov.br/bvs/saudelegis/gm/2010/prt4279_30_12_2010.html

36. Brasil. Ministério da Saúde. Portaria no 2.488, de 21 de outubro de 2011 [Internet]. 2488 out 21, 2011. Disponível em: https://bvsms.saude.gov.br/bvs/saudelegis/gm/2011/prt2488_21_10_2011.html

37. Brasil. Ministério da Saúde. Secretaria de Atenção à Saúde. Departamento de Atenção Básica. Política Nacional de Atenção Básica [Internet]. Brasília: Ministério da Saúde; 2012 [citado 17 de fevereiro de 2022] p. 114. Disponível em: http://189.28.128.100/dab/docs/publicacoes/geral/pnab.pdf

38. Brasil. Ministério da Saúde. Portaria no 2.715, de 17 de novembro de 2011 [Internet]. 2715 nov 17, 2011. Disponível em: https://bvsms.saude.gov.br/bvs/saudelegis/gm/2011/prt2715_17_11_2011.html

39. Brasil. Ministério da Saúde. Secretária de Atenção à Saúde. Departamento de Atenção Básica. Política Nacional de Alimentação e Nutrição [Internet]. Brasília: Ministério da Saúde; 2011 [citado 4 de setembro de 2022]. Disponível em: https://bvsms.saude.gov.br/bvs/publicacoes/politica_nacional_alimentacao_nutricao.pdf

40. Brasil. Ministério da Saúde. Secretária de Atenção à Saúde. Departamento de Atenção Básica. Orientações para a coleta e análise de dados antropométricos em serviços de saúde: norma técnica do Sistema de Vigilância Alimentar e Nutricional - SISVAN [Internet]. Ministério da Saúde; 2011 [citado 4 de setembro de 2022]. Disponível em: https://bvsms.saude.gov.br/bvs/publicacoes/orientacoes_coleta_analise_dados_antropometricos.pdf

41. Brasil. Ministério da Saúde. Decreto no 7.508, de 28 de junho de 2011 [Internet]. 7508 jun 28, 2011. Disponível em: http://www.planalto.gov.br/ccivil_03/_ato2011-2014/2011/decreto/d7508.htm

42. Brasil. Ministério da Saúde. Portaria no 2.975, de 14 de dezembro de 2011 [Internet]. 2975 dez 14, 2011. Disponível em: https://bvsms.saude.gov.br/bvs/saudelegis/gm/2011/prt2975_14_12_2011.html

43. Brasil. Ministério da Saúde. Secretaria de Atenção à Saúde. Departamento de Atenção Básica. Coordenação-Geral de Alimentação e Nutrição. Manual orientador para aquisição de equipamentos antropométricos [Internet]. Ministério da Saúde; 2012 [citado 4 de setembro de 2022]. Disponível em: http://189.28.128.100/dab/docs/portaldab/publicacoes/manual_equipamentos_2012_1201.pdf

44. Brasil. Ministério da Saúde. Portaria no 2.387, de 18 de outubro de 2012 [Internet]. 2387 out 18, 2012. Disponível em: https://bvsms.saude.gov.br/bvs/saudelegis/gm/2012/prt2387_18_10_2012.html

45. Brasil. Ministério da Saúde. Secretaria de Atenção à Saúde. Departamento de Atenção Básica. Manual instrutivo para implementação da Agenda para Intensificação da Atenção

Nutricional Infantil: Portaria no 2.387, de 18 de outubro de 2012 [Internet]. Ministério da Saúde; 2013 [citado 4 de setembro de 2022]. Disponível em: http://189.28.128.100/dab/docs/portaldab/publicacoes/manual_andi.pdf

46. Brasil. Ministério da Saúde. Portaria no 2.446, de 11 de novembro de 2014 [Internet]. 2446 nov 11, 2014. Disponível em: https://bvsms.saude.gov.br/bvs/saudelegis/gm/2014/prt2446_11_11_2014.html

47. Brasil. Ministério da Saúde. Secretaria de Atenção à Saúde. Departamento de Atenção Básica. Marco de referência da vigilância alimentar e nutricional na atenção básica [Internet]. Ministério da Saúde; 2015 [citado 4 de setembro de 2022]. Disponível em: http://189.28.128.100/dab/docs/portaldab/publicacoes/marco_referencia_vigilancia_alimentar.pdf

48. Brasil. Ministério da Saúde. Secretaria de Atenção à Saúde. Departamento de Atenção Básica. Orientações para avaliação de marcadores de consumo alimentar na atenção básica [Internet]. Brasília: Ministério da Saúde; 2015 [citado 21 de fevereiro de 2022] p. 33. Disponível em: http://189.28.128.100/dab/docs/portaldab/publicacoes/marcadores_consumo_alimentar_atencao_basica.pdf

49. Brasil. Ministério da Saúde. Portaria no 2.436, de 21 de setembro de 2017 [Internet]. 2436 set 21, 2017. Disponível em: https://bvsms.saude.gov.br/bvs/saudelegis/gm/2017/prt2436_22_09_2017.html

50. Brasil. Ministério da Saúde. Manual operacional para uso do sistema de vigilância alimentar e nutricional. SISVAN - versão 3.0. Versão preliminar [Internet]. Brasília: Ministério da Saúde; 2017 [citado 4 de setembro de 2022] p. 39. Disponível em: http://sisaps.saude.gov.br/sisvan/public/file/ManualDoSisvan.pdf

51. Brasil. Ministério da Saúde. Secretaria de Vigilância em Saúde. Secretaria de Atenção à Saúde. Política Nacional de Promoção da Saúde: PNPS: Anexo I da Portaria de Consolidação no 2, de 28 de setembro de 2017, que consolida as normas sobre as políticas nacionais de saúde do SUS [Internet]. Brasília: Ministério da Saúde; 2018 [citado 17 de fevereiro de 2022] p. 42. Disponível em: https://bvsms.saude.gov.br/bvs/publicacoes/politica_nacional_promocao_saude.pdf

52. Conselho Nacional de Saúde. Resolução no 588, de 12 de julho de 2018 [Internet]. 588 jul 12, 2022. Disponível em: http://conselho.saude.gov.br/resolucoes/2018/Reso588.pdf

53. Brasil. Ministério da Saúde. Secretaria de Atenção à Saúde. Secretaria de Vigilância em Saúde. Guia Política Nacional de Atenção Básica - Módulo 1: Integração Atenção Básica e Vigilância em Saúde [Internet]. Ministério da Saúde; 2018 [citado 4 de setembro de 2022]. Disponível em: http://189.28.128.100/dab/docs/portaldab/publicacoes/guia_pnab.pdf

54. Brasil. Ministério da Saúde. Portaria no 1.862, de 10 de agosto de 2021 [Internet]. 1862 ago 10, 2021. Disponível em: https://www.in.gov.br/en/web/dou/-/portaria-gm/ms-n-1.862-de-10-de-agosto-de-2021-337532485

55. Brasil. Ministério da Saúde. Secretaria de Atenção Primária à Saúde. Departamento de Promoção da Saúde. PROTEJA: Estratégia Nacional para Prevenção e Atenção à Obesidade Infantil: orientações técnicas [versão preliminar] [Internet]. Ministério da Saúde; 2021 [citado 4 de setembro de 2022]. Disponível em: http://189.28.128.100/dab/docs/portaldab/publicacoes/orienta_proteja.pdf

56. Brasil. Ministério da Saúde. Universidade do Estado do Rio de Janeiro. Instrutivo para o cuidado da criança e do adolescente com sobrepeso e obesidade no âmbito da Atenção Primária à Saúde [versão preliminar] [Internet]. Ministério da Saúde; 2021 [citado 4 de setembro de 2022]. Disponível em: http://189.28.128.100/dab/docs/portaldab/publicacoes/instrutivo_crianca_adolescente.pdf

57. Brasil. Presidência da República. Lei no 14.284, de 29 de dezembro de 2021 [Internet]. 14284 dez 29, 2021. Disponível em: https://www.in.gov.br/en/web/dou/-/lei-n-14.284-de-29-de-dezembro-de-2021-370918498

58. Brasil. Ministério da Saúde. Secretaria de Atenção Primária à Saúde. Departamento de Promoção da Saúde. Matriz para Organização dos Cuidados em Alimentação e Nutrição na Atenção Primária à Saúde [versão preliminar] [Internet]. Brasília: Ministério da Saúde; 2022 [citado 15 de setembro de 2022] p. 95. Disponível em: http://189.28.128.100/dab/docs/portaldab/publicacoes/matriz_organizacao_cuidados_nutricao_aps.pdf

59. Brasil. Ministério da Saúde. Universidade Federal de Sergipe. Guia para a Organização da Vigilância Alimentar e Nutricional na Atenção Primária à Saúde [Internet]. Brasília: Ministério da Saúde; 2022 [citado 11 de dezembro de 2022]. 51 p. Disponível em: https://bvsms.saude.gov.br/bvs/publicacoes/guia_organizacao_vigilancia_alimentar_nutricional.pdf

Quadro S2 Fontes de dados disponíveis para a vigilância alimentar e nutricional no Brasil, 1974 a 2022.

FONTE DE DADOS	OBJETIVOS	ABRANGÊNCIA	TAMANHO DE AMOSTRA	DADOS DISPONÍVEIS: ANTROPOMETRIA, CONSUMO ALIMENTAR, DISPONIBILIDADE ALIMENTAR E/OU SEGURANÇA ALIMENTAR E NUTRICIONAL
Estudo Nacional da Despesa Familiar – ENDEF 1974-1975[1]	Obter um fluxo mais completo de estatísticas sociais.	Nacional, à exceção das áreas rurais das regiões Norte e Centro-Oeste	n≈55000 (domicílios)	Antropometria, consumo alimentar e disponibilidade alimentar
Pesquisa Nacional sobre Saúde Materno-Infantil e Planejamento Familiar – PNSMIPF 1986[2]	Criar uma referência mais precisa, no processo de conhecimento da realidade nacional, nas áreas de saúde materno-infantil, reprodução humana e planejamento familiar.	Nacional, à exceção das áreas rurais das Regiões Norte e Centro-Oeste, Acre, territórios de Rondônia, Roraima e Amapá	n=8408	Antropometria (Região Nordeste) e consumo alimentar
Pesquisa de Orçamentos Familiares – POF 1987-1988[3]	Mensurar as estruturas dos gastos, receitas e poupanças das famílias, ou seja, são observadas as condições de vida das famílias a partir da análise dos orçamentos.	Regiões Metropolitanas de Belém, Fortaleza, Recife, Salvador, Belo Horizonte, Rio de Janeiro, São Paulo, Curitiba, Porto Alegre, Brasília-DF, município de Goiânia	Não disponível	Disponibilidade alimentar
Pesquisa Nacional sobre Saúde e Nutrição – PNSN 1989[4]	Produzir informações sobre o estado nutricional e de saúde da população brasileira, incluindo dados antropométricos, condições de saúde, acesso a serviços públicos de saúde, suplementação alimentar e dados obstétricos.	Nacional, à exceção da área rural da Região Norte	n=17920 (domicílios)	Antropometria e consumo alimentar
Pesquisa sobre Saúde Familiar no Nordeste Brasil – PSFNe 1991[5]	Fornecer informações sobre os níveis atuais de fecundidade e mortalidade infantil, conhecimento e uso da anticoncepção, intenções reprodutivas, demanda de anticoncepção, planejamento da fecundidade, uso de serviços de saúde materno-infantil, nutrição infantil, imunização, tratamento de doenças infantis e conhecimento sobre doenças sexualmente transmissíveis/AIDS.	Região Nordeste	n=6064	Consumo alimentar
Pesquisa de Orçamentos Familiares – POF 1995-1996[6]	Mensurar as estruturas dos gastos, receitas e poupanças das famílias, ou seja, são observadas as condições de vida das	Regiões Metropolitanas de Belém, Fortaleza, Recife, Salvador, Belo Horizonte, Rio de Janeiro, São Paulo,	n=16014 (domicílios)	Disponibilidade alimentar

	famílias a partir da análise dos orçamentos.	Curitiba, Porto Alegre, Brasília-DF, município de Goiânia		
Pesquisa Nacional sobre Demografia e Saúde – PNDS 1996[7]	Levantar informações atualizadas sobre os níveis de fecundidade, mortalidade infantil e materna, anticoncepção, saúde da mulher e da criança, conhecimento e atitudes relacionadas às DST/AIDS.	Nacional	n=13283 (domicílios)	Antropometria e consumo alimentar
Pesquisa sobre Padrões de Vida – PPV 1996-1997[8]	Qualificar e indicar os determinantes do bem-estar social de diferentes grupos sociais; e identificar os efeitos de políticas governamentais nas condições de vida domiciliar.	Regiões Nordeste e Sudeste	n≈5000 (domicílios)	Antropometria e disponibilidade alimentar
Estudo Multicêntrico sobre Consumo Alimentar 1997[9]	Avaliar metodologias de inquérito dietético e trazer informações novas sobre o consumo alimentar da população.	Campinas, Curitiba, Goiânia, Ouro Preto, Rio de Janeiro	n=7972	Consumo alimentar e disponibilidade alimentar
I Pesquisa de Prevalência de Aleitamento Materno nas Capitais Brasileiras e Distrito Federal 1999[10]	Verificar a situação atual da amamentação e da alimentação complementar no Brasil.	Capitais de 25 estados brasileiros e o Distrito Federal	n=48845	Consumo alimentar
Pesquisa de Orçamentos Familiares – POF 2002-2003[11]	Atualizar as estruturas de ponderações, necessárias para a produção dos índices de Preços ao Consumidor e também na atualização da participação das despesas das famílias no cálculo das Contas Nacionais.	Nacional	n=48470 (domicílios)	Antropometria e disponibilidade alimentar
Pesquisa Nacional por Amostra de Domicílios: Segurança Alimentar – PNAD 2004[12]	Descrever a condição de segurança alimentar nos domicílios brasileiros com base em metodologia que permite também detectar e dimensionar os problemas de insegurança alimentar.	Nacional	n=139157 (domicílios)	Segurança alimentar e nutricional
Chamada Nutricional de crianças menores de 5 anos de idade residentes no Semiárido e assentamentos da Região Nordeste e do Norte de Minas Gerais 2005[13]	Avaliar a situação nutricional das crianças menores de cinco anos.	Semiárido e assentamentos da Região Nordeste e do Norte de Minas Gerais	Semiárido (n=16239), assentamentos (n=1305)	Antropometria e consumo alimentar
Chamada Nutricional para crianças menores de cinco anos de idade no Estado do Amazonas 2006[14]	Avaliar a situação nutricional das crianças menores de cinco anos, residentes na área urbana dos municípios amazonenses bem como identificar aquelas com déficit nutricional e encaminhá-las para atendimento de saúde e acompanhamento social.	Estado do Amazonas	n=4646	Antropometria e consumo alimentar
Chamada Nutricional Quilombola 2006[15]	Caracterizar a situação sociodemográfica das	60 comunidades quilombolas de 22	n=2941	Antropometria e consumo alimentar

	famílias e o estado nutricional de crianças menores de cinco anos pertencentes a comunidades remanescentes de quilombos.	unidades da federação		
Pesquisa Nacional de Demografia e Saúde da Criança e da Mulher – PNDS 2006[16]	Caracterizar a população feminina em idade fértil e as crianças menores de cinco anos segundo fatores demográficos, socioeconômicos e culturais; identificar padrões de conjugalidade, parentalidade e reprodutivos; identificar perfis de morbimortalidade na infância e de amamentação; avaliar o estado nutricional; avaliar a segurança/insegurança alimentar; o teor de iodo disponível em âmbito domiciliar e avaliar o acesso a serviços de saúde e a medicamentos.	Nacional	n=14617 (domicílios)	Antropometria, consumo alimentar, disponibilidade alimentar (sal iodado) e segurança alimentar e nutricional
Vigilância de fatores de risco e proteção para doenças crônicas por inquérito telefônico 2006-atual[17]	Monitorar a frequência e a distribuição de fatores de risco e proteção para doenças crônicas não transmissíveis.	Capitais dos 26 estados brasileiros e o Distrito Federal	O sistema estabelece um tamanho amostral mínimo de ~2000 entrevistados por cidade	Antropometria (referido) e consumo alimentar
Chamada Nutricional da Região Norte 2007[18]	Obter informações mais detalhadas das condições nutricionais da população infantil da região Norte.	Estados da Região Norte	n=14418	Antropometria e consumo alimentar
II Pesquisa de Prevalência de Aleitamento Materno nas Capitais Brasileiras e Distrito Federal 2008[19]	Verificar a situação atual da amamentação e da alimentação complementar no Brasil, analisar a evolução dos indicadores de aleitamento materno no período de 1999 a 2008, identificar grupos populacionais mais vulneráveis à interrupção do aleitamento materno e avaliar práticas alimentares saudáveis e não saudáveis.	Capitais dos 26 estados brasileiros e o Distrito Federal	n=34366	Consumo alimentar
I Inquérito Nacional de Saúde e Nutrição dos Povos Indígenas 2008-2009[20]	Caracterizar o estado nutricional e de saúde de crianças menores de 5 anos e de mulheres de 14 a 49 anos.	Nacional	Mulheres (n=6692), crianças (n=6128)	Antropometria, consumo alimentar e disponibilidade alimentar
Pesquisa de Orçamentos Familiares – POF 2008-2009[21]	Fornecer informações sobre a composição dos orçamentos domésticos, a partir da investigação dos hábitos de consumo, da alocação de gastos e da distribuição dos rendimentos, segundo as características dos	Nacional	n=55970 (domicílios)	Antropometria, consumo alimentar (≥10 anos) e disponibilidade alimentar

	domicílios e das pessoas. Investigar a autopercepção da qualidade de vida e as características do perfil nutricional da população brasileira.			
Sistema de Vigilância Alimentar e Nutricional (SISVAN) 2008-atual[22]	Consolidar os dados referentes às ações de Vigilância Alimentar e Nutricional, desde o registro de dados antropométricos e de marcadores de consumo alimentar até a geração de relatórios.	Nacional	Não disponível	Antropometria (2008-atual) e consumo alimentar (2015-atual)
Pesquisa Nacional de Saúde do Escolar – PENSE 2009[23]	Produzir informações que permitam conhecer a magnitude dos fatores de risco e proteção à saúde, orientar e avaliar as intervenções em saúde adequadas ao adolescente, contribuindo para o monitoramento da saúde do escolar.	Capitais dos 26 estados brasileiros e o Distrito Federal	n=60973	Antropometria e consumo alimentar
Pesquisa Nacional por Amostra de Domicílios: Segurança Alimentar – PNAD 2009[24]	Descrever a condição de segurança alimentar nos domicílios brasileiros com base em metodologia que permite também detectar e dimensionar os problemas de insegurança alimentar.	Nacional	n=153837 (domicílios)	Segurança alimentar e nutricional
Pesquisa Nacional de Saúde do Escolar – PENSE 2012[25]	Produzir informações que permitam conhecer a magnitude dos fatores de risco e proteção à saúde, orientar e avaliar as intervenções em saúde adequadas ao adolescente, contribuindo para o monitoramento da saúde do escolar.	Nacional	n=109104	Antropometria (referido) e consumo alimentar
Pesquisa Nacional de Saúde – PNS 2013[26]	Coletar informações sobre o desempenho do sistema nacional de saúde no que se refere ao acesso e uso dos serviços disponíveis e à continuidade dos cuidados, bem como sobre as condições de saúde da população, a vigilância de doenças crônicas não transmissíveis e os fatores de risco a elas associados.	Nacional	n=62658 (domicílios)	Antropometria e consumo alimentar
Pesquisa Nacional por Amostra de Domicílios: Segurança Alimentar – PNAD 2013[27]	Descrever a condição de segurança alimentar nos domicílios brasileiros com base em metodologia que permite também detectar e dimensionar os problemas de	Nacional	n=148697 (domicílios)	Segurança alimentar e nutricional

	insegurança alimentar.			
Estudo de Riscos Cardiovasculares em Adolescentes – ERICA 2013-2014[28]	Estimar a prevalência de diabetes mellitus, obesidade, fatores de risco cardiovascular e de marcadores de resistência à insulina e inflamatórios em adolescentes de 12 a 17 anos que frequentem escolas com mais de 100000 habitantes. Com base nesses achados, também foi estimada a prevalência de síndrome metabólica.	Nacional	n=74589	Antropometria e consumo alimentar
Pesquisa Nacional de Saúde do Escolar – PENSE 2015[29]	Produzir informações que permitam conhecer a magnitude dos fatores de risco e proteção à saúde, orientar e avaliar as intervenções em saúde adequadas ao adolescente, contribuindo para o monitoramento da saúde do escolar.	Nacional	Amostra 1 (n=102072), amostra 2 (n=10926)	Antropometria (referido na amostra 1 e aferido e referido na amostra 2) e consumo alimentar
Pesquisa de Orçamentos Familiares – POF 2017-2018[30]	A POF visa, principalmente, mensurar as estruturas de consumo, gastos, dos rendimentos e parte da variação patrimonial das famílias.	Nacional	n=57920 [domicílios]	Antropometria (referido, indivíduos ≥10 anos) e consumo alimentar (≥10 anos), disponibilidade alimentar e segurança alimentar e nutricional
Estudo Nacional de Alimentação e Nutrição Infantil – ENANI 2019[31]	Avaliar as práticas de aleitamento materno e de alimentação complementar, o consumo alimentar, o estado nutricional antropométrico infantil e durante a gestação e a deficiência de micronutrientes (hemoglobina, vitamina A, vitamina D, vitamina E, vitamina B1, vitamina B6, vitamina B12, folato, zinco, selênio e ferritina) entre crianças brasileiras menores de 5 anos.	Nacional	n=12524 (domicílios)	Antropometria, consumo alimentar e segurança alimentar e nutricional
Pesquisa Nacional de Saúde – PNS 2019[32]	Coletar informações sobre o desempenho do sistema nacional de saúde no que se refere ao acesso e uso dos serviços disponíveis e à continuidade dos cuidados, bem como sobre as condições de saúde da população, a vigilância de doenças crônicas não transmissíveis e os fatores de risco a elas associados.	Nacional	n=90846 (domicílios)	Antropometria e consumo alimentar
Pesquisa Nacional de Saúde do Escolar –	Produzir informações que permitam conhecer	Nacional	n=125123	Consumo alimentar

PENSE 2019[33]	a magnitude dos fatores de risco e proteção à saúde, orientar e avaliar as intervenções em saúde adequadas ao adolescente, contribuindo para o monitoramento da saúde do escolar.			

Referências

1. Instituto Brasileiro de Geografia e Estatística. Estudo Nacional da Despesa Familiar: Consumo Alimentar e Antropometria [Internet]. Rio de Janeiro; 1977 [citado 28 de agosto de 2022] p. 100. Disponível em: http://189.28.128.100/dab/docs/portaldab/documentos/endef_consumo_alimentar_antropometria_regiao2_regiao4.pdf

2. Sociedade Civil Bem-Estar Familiar no Brasil (BEMFAM). Pesquisas Demográficas e de Saúde. Instituto para Desenvolvimento de Recursos (IRD). Pesquisa Nacional sobre Saúde Materno-Infantil e Planejamento Familiar - 1986 [Internet]. Rio de Janeiro: Sociedade Civil Bem-Estar Familiar no Brasil; 1987 [citado 4 de setembro de 2022] p. 255. Disponível em: https://dhsprogram.com/pubs/pdf/FR4/FR4.pdf

3. Instituto Brasileiro de Geografia e Estatística. IBGE – Orçamento Familiar: Pesquisa de Orçamentos Familiares [Internet]. [citado 4 de setembro de 2022]. Disponível em: https://ces.ibge.gov.br/apresentacao/portarias/200-comite-de-estatisticas-sociais/base-de-dados/1145-pesquisa-de-orcamentos-familiares.html

4. Instituto Nacional de Alimentação e Nutrição (INAN). Instituto Brasileiro de Geografia e Estatística (IBGE). Instituto de Planejamento Econômico e Social (IPEA). Pesquisa Nacional sobre Saúde e Nutrição: Resultados Preliminares (PNSN 1989) [Internet]. 1990 nov [citado 4 de setembro de 2022] p. 30. Disponível em: https://biblioteca.ibge.gov.br/visualizacao/livros/liv81879.pdf

5. Sociedade Civil Bem-Estar Familiar no Brasil (BEMFAM). Pesquisa sobre Saúde Familiar no Nordeste Brasil 1991 [Internet]. Sociedade Civil Bem-Estar Familiar no Brasil; 1992 out [citado 4 de setembro de 2022] p. 273. Disponível em: https://dhsprogram.com/pubs/pdf/FR5/FR5.pdf

6. Instituto Brasileiro de Geografia e Estatística. Pesquisa de Orçamentos Familiares 1995-1996: Primeiros Resultados [Internet]. Rio de Janeiro: Instituto Brasileiro de Geografia e Estatística; 1997 [citado 4 de setembro de 2022] p. 217. Disponível em: https://biblioteca.ibge.gov.br/visualizacao/livros/liv7924.pdf

7. Sociedade Civil Bem-Estar Familiar no Brasil (BEMFAM). Pesquisa Nacional sobre Demografia e Saúde 1996 [Internet]. Sociedade Civil Bem-Estar Familiar no Brasil; 1997 [citado 4 de setembro de 2022] p. 296. Disponível em: https://dhsprogram.com/pubs/pdf/FR77/FR77.pdf

8. Instituto Brasileiro de Geografia e Estatística. Pesquisa sobre Padrões de Vida 1996-1997 [Internet]. Rio de Janeiro: Instituto Brasileiro de Geografia e Estatística; 1999 [citado 4 de setembro de 2022] p. 141. Disponível em: https://biblioteca.ibge.gov.br/visualizacao/livros/liv6642.pdf

9. Brasil. Ministério da Saúde. Estudo Multicêntrico sobre Consumo Alimentar - 1997 [Internet]. Ministério da Saúde; 1997 [citado 4 de setembro de 2022] p. 57. Disponível em: https://bvsms.saude.gov.br/bvs/publicacoes/cadernosespecial.pdf

10. Brasil. Ministério da Saúde. Pesquisa de Prevalência do Aleitamento Materno nas Capitais e no Distrito Federal [Internet]. Brasília: Ministério da Saúde; 2001 [citado 4 de setembro de 2022] p. 121. Disponível em: https://bvsms.saude.gov.br/bvs/publicacoes/pesquisa_prevalencia_aleitamento_materno_2001.pdf

11. Instituto Brasileiro de Geografia e Estatística. Pesquisa de Orçamentos Familiares 2002-2003: análise da disponibilidade domiciliar de alimentos e do estado nutricional no Brasil [Internet]. Rio de Janeiro: Instituto Brasileiro de Geografia e Estatística; 2004 [citado 4 de setembro de 2022] p. 76. Disponível em: https://biblioteca.ibge.gov.br/visualizacao/livros/liv4472.pdf

12. Instituto Brasileiro de Geografia e Estatística. Pesquisa Nacional por Amostra de Domicílios: Segurança Alimentar 2004 [Internet]. Rio de Janeiro: Instituto Brasileiro de Geografia e Estatística; 2006 [citado 21 de fevereiro de 2022] p. 140. Disponível em: https://biblioteca.ibge.gov.br/visualizacao/livros/liv29775.pdf

13. Brasil. Ministério do Desenvolvimento Social e Combate à Fome. Secretaria de Avaliação e Gestão da Informação. Chamada nutricional em crianças menores de cinco anos em situação de vulnerabilidade social no Semiárido e assentamentos rurais do Nordeste [Internet]. Brasília: Ministério do Desenvolvimento Social e Combate à Fome; 2006 abr [citado 4 de setembro de 2022] p. 6. Disponível em: http://aplicacoes.mds.gov.br/sagi/pesquisas/documentos/PainelPEI/Publicacoes/Chamada%20Nutricional%20em%20crian%C3%A7as%20menores%20de%20cinco%20anos%20em%20situa%C3%A7%C3%A3o%20de%20vulnerabilidade%20social%20no%20Semi%C3%A1rido%20e%20assentamentos%20rurais%20do%20Nordeste.pdf

14. Governo do Estado do Amazonas. Secretaria de Estado da Saúde do Amazonas. Ministério do Desenvolvimento Social e Combate à Fome. Secretaria de Avaliação e Gestão da Informação. Ministério da Saúde. Instituto Nacional de Pesquisa da Amazônia. Chamada nutricional para crianças menores de cinco anos de idade no estado do Amazonas, 2006 [Internet]. Ministério do Desenvolvimento Social e Combate à Fome; 2007 set [citado 4 de setembro de 2022] p. 14. Disponível em: http://aplicacoes.mds.gov.br/sagi/pesquisas/documentos/PainelPEI/Publicacoes/Sum%C3%A1rio%20Executivo%20Chamada%20Nutricional%20Amazonas.pdf

15. Brasil. Ministério do Desenvolvimento Social e Combate à Fome. Secretaria de Avaliação e Gestão da Informação. Departamento de Avaliação e Monitoramento. Chamada Nutricional Quilombola [Internet]. Ministério do Desenvolvimento Social e Combate à Fome; 2007 maio [citado 4 de setembro de 2022] p. 8. Disponível em: https://fpabramo.org.br/acervosocial/wp-content/uploads/sites/7/2017/08/352.pdf

16. Brasil. Ministério da Saúde. Pesquisa Nacional de Demografia e Saúde da Criança e da Mulher - PNDS 2006: dimensões do processo reprodutivo e da saúde da criança [Internet]. Brasília: Ministério da Saúde; 2009 [citado 14 de dezembro de 2021] p. 302. Disponível em: https://bvsms.saude.gov.br/bvs/publicacoes/pnds_crianca_mulher.pdf

17. Brasil. Ministério da Saúde. VIGITEL Brasil 2006. Vigilância de fatores de risco e proteção para doenças crônicas por inquérito telefônico. Estimativas sobre frequência e distribuição sociodemográfica de fatores de risco e proteção para doenças crônicas nas capitais dos 26 estados brasileiros e no Distrito Federal em 2006 [Internet]. Brasília: Ministério da Saúde; 2007 [citado 4 de

setembro de 2022] p. 298. Disponível em: https://bvsms.saude.gov.br/bvs/publicacoes/vigitel_brasil_2006.pdf

18. Brasil. Ministério da Saúde. Secretaria de Atenção à Saúde. Departamento de Atenção Básica. Coordenação-Geral da Política de Alimentação e Nutrição. Chamada Nutricional da Região Norte 2007 [Internet]. Brasília: Ministério da Saúde; 2009 mar [citado 4 de setembro de 2022] p. 18. Disponível em: http://189.28.128.100/dab/docs/portaldab/documentos/chamada_nutricional_regiao_norte.pdf

19. Brasil. Ministério da Saúde. Secretaria de Atenção à Saúde. Departamento de Ações Programáticas e Estratégicas. II Pesquisa de Prevalência de Aleitamento Materno nas Capitais Brasileiras e Distrito Federal [Internet]. Brasília: Ministério da Saúde; 2009 [citado 21 de fevereiro de 2022] p. 108. Disponível em: https://bvsms.saude.gov.br/bvs/publicacoes/pesquisa_prevalencia_aleitamento_materno.pdf

20. Coimbra CE, Santos RV, Welch JR, Cardoso AM, de Souza MC, Garnelo L, et al. The First National Survey of Indigenous People's Health and Nutrition in Brazil: rationale, methodology, and overview of results. BMC Public Health. 19 de janeiro de 2013;13(1):52.

21. Instituto Brasileiro de Geografia e Estatística (IBGE). Pesquisa de Orçamentos Familiares 2008-2009. Antropometria e estado nutricional de crianças, adolescentes e adultos no Brasil. [Internet]. Rio de Janeiro: Instituto Brasileiro de Geografia e Estatística; 2010 [citado 21 de fevereiro de 2022]. Disponível em: http://www.ibge.gov.br/home/estatistica/populacao/condicaodevida/pof/2008_2009/POFpublicacao.pdf

22. Brasil. Ministério da Saúde. Sistema de Vigilância Alimentar e Nutricional - SISVAN [Internet]. [citado 4 de setembro de 2022]. Disponível em: https://sisaps.saude.gov.br/sisvan/

23. Instituto Brasileiro de Geografia e Estatística. Ministério da Saúde. Pesquisa Nacional de Saúde do Escolar 2009 [Internet]. Rio de Janeiro: Instituto Brasileiro de Geografia e Estatística; 2009 [citado 4 de setembro de 2022] p. 138. Disponível em: https://biblioteca.ibge.gov.br/visualizacao/livros/liv43063.pdf

24. Instituto Brasileiro de Geografia e Estatística. Ministério do Desenvolvimento Social e Combate à Fome. Pesquisa Nacional por Amostra de Domicílios: Segurança Alimentar 2004/2009 [Internet]. Rio de Janeiro: Instituto Brasileiro de Geografia e Estatística; 2010 [citado 4 de setembro de 2022] p. 183. Disponível em: https://biblioteca.ibge.gov.br/visualizacao/livros/liv47241.pdf

25. Instituto Brasileiro de Geografia e Estatística. Ministério da Saúde. Pesquisa Nacional de Saúde do Escolar 2012 [Internet]. Rio de Janeiro: Instituto Brasileiro de Geografia e Estatística; 2013 [citado 4 de setembro de 2022] p. 256. Disponível em: https://biblioteca.ibge.gov.br/visualizacao/livros/liv64436.pdf

26. Instituto Brasileiro de Geografia e Estatística. Ministério da Saúde. Fundação Oswaldo Cruz. Pesquisa Nacional de Saúde: 2013: Ciclos de vida: Brasil e grandes regiões [Internet]. Rio de Janeiro: Instituto Brasileiro de Geografia e Estatística; 2015 [citado 4 de setembro de 2022] p. 90. Disponível em: https://biblioteca.ibge.gov.br/visualizacao/livros/liv94522.pdf

27. Instituto Brasileiro de Geografia e Estatística. Ministério do Desenvolvimento Social e Combate à Fome. Pesquisa Nacional por Amostra de Domicílios: Segurança Alimentar 2013

[Internet]. Rio de Janeiro: Instituto Brasileiro de Geografia e Estatística; 2014 [citado 4 de setembro de 2022] p. 134. Disponível em: https://biblioteca.ibge.gov.br/visualizacao/livros/liv91984.pdf

28. da Silva TLN, Klein CH, Souza A de M, Barufaldi LA, Abreu G de A, Kuschnir MCC, et al. Response rate in the Study of Cardiovascular Risks in Adolescents - ERICA. Rev Saude Publica. fevereiro de 2016;50 Suppl 1(Suppl 1):3s.

29. Instituto Brasileiro de Geografia e Estatística. Ministério da Saúde. Pesquisa Nacional de Saúde do Escolar 2015 [Internet]. Brasília: Instituto Brasileiro de Geografia e Estatística; 2016 [citado 4 de setembro de 2022] p. 131. Disponível em: https://biblioteca.ibge.gov.br/visualizacao/livros/liv97870.pdf

30. Instituto Brasileiro de Geografia e Estatística. Pesquisa de Orçamentos Familiares 2017-2018: Avaliação nutricional da disponibilidade domiciliar de alimentos no Brasil [Internet]. Rio de Janeiro: Instituto Brasileiro de Geografia e Estatística; 2020 [citado 21 de fevereiro de 2022] p. 61. Disponível em: https://biblioteca.ibge.gov.br/visualizacao/livros/liv101704.pdf

31. Alves-Santos NH, Castro IRR de, Anjos LAD, Lacerda EM de A, Normando P, Freitas MB de, et al. General methodological aspects in the Brazilian National Survey on Child Nutrition (ENANI-2019): a population-based household survey. Cad Saude Publica. 2021;37(8):e00300020.

32. Instituto Brasileiro de Geografia e Estatística. Ministério da Saúde. Pesquisa Nacional de Saúde: 2019. Atenção Primária à Saúde e Informações Antropométricas [Internet]. Rio de Janeiro: Instituto Brasileiro de Geografia e Estatística; 2020 [citado 28 de agosto de 2022] p. 70. Disponível em: https://biblioteca.ibge.gov.br/visualizacao/livros/liv101758.pdf

33. Instituto Brasileiro de Geografia e Estatística. Ministério da Saúde. Pesquisa Nacional de Saúde do Escolar: 2019 [Internet]. Rio de Janeiro; 2021 [citado 22 de fevereiro de 2022]. 162 p. Disponível em: https://biblioteca.ibge.gov.br/visualizacao/livros/liv101852.pdf

Quadro S3 Documentos relacionados à vigilância alimentar e nutricional em Portugal, 1980 a 2022.

DOCUMENTO	TIPO	SÍNTESE
Decreto-lei nº 265/80, de 7 de Agosto[1]	Decreto-lei	Criação do Conselho de Alimentação e Nutrição (CAN) que tinha entre os seus objetivos a formulação dos princípios orientadores de uma política de alimentação e nutrição.
Decreto-lei nº 278/84, de 10 de Agosto[2]	Decreto-lei	Criação do Conselho Nacional de Alimentação e Nutrição (CNAN) em substituição ao Conselho de Alimentação e Nutrição (CAN).
Tratado da União Europeia – Tratado de Maastricht[3]	Tratado	Início efetivo de ações articuladas na área de saúde pública na comunidade europeia.
Tratado da União Europeia [alterações introduzidas pelo Tratado de Amsterdã][4]	Tratado	A revisão do Tratado declara a necessidade de assegurar a melhoria da saúde na elaboração e implementação de políticas públicas.
Core report Eurodiet: Nutrition & Diet for Healthy Lifestyles in Europe[5]	Relatório	O projeto "Eurodiet" analisou as relações entre saúde e nutrientes, para posterior produção de guias baseados em alimentos para a promoção de estilos de vida saudáveis. O relatório aponta a necessidade de métodos harmonizados de vigilância alimentar e nutricional na União Europeia.
The First Action Plan for Food and Nutrition Policy (2000-2005)[6]	Plano de Ação	O documento indica a necessidade do desenvolvimento de políticas alimentar e nutricional que protejam e promovam a saúde e reduzam a carga de doenças relacionadas com a alimentação, enquanto possam contribuir para o desenvolvimento socioeconômico e para um ambiente sustentável. Em relação à vigilância, encoraja-se o desenvolvimento de sistemas com indicadores custo-efetivos para ajudar os países a avaliar as suas políticas. Nesse Plano de Ação há uma vertente importante para a vigilância de doenças transmitidas por alimentos.
Food and nutrition action plans in the WHO European Region – Past, present and future[7]	Relatório	O objetivo do relatório foi documentar o encontro realizado em Atenas, comunicar as discussões realizadas pelos grupos individuais e reportar as recomendações resultantes do encontro. O relatório destaca que a vigilância e o monitoramento são essenciais para o Plano de Ação.
Plano Nacional de Saúde: Prioridades para 2004-2010[8]	Política	O Plano Nacional de Saúde contém orientações estratégicas para um conjunto mínimo de atividades que as instituições ligadas ao Ministério da Saúde devem assegurar para ganhos em saúde e em eficiência, orientado para a prevenção de doenças e enfermidades, nas suas vertentes primárias, secundárias e terciárias. O Plano realça a importância de adotar uma estratégia de incentivo à investigação e desenvolvimento em saúde.
Programa Nacional de Intervenção Integrada sobre Determinantes de Saúde Relacionados com os Estilos de Vida[9]	Programa	O Programa insere-se no Plano Nacional de Saúde e tem como foco o consumo de tabaco, a alimentação, o consumo de álcool, a atividade física e a gestão de estresse. O Programa tem como objetivo geral reduzir a prevalência de fatores de risco de doenças crônicas não transmissíveis e aumentar a prevalência de

		fatores de proteção, relacionados com os estilos de vida, através de uma abordagem integrada e intersetorial. O Programa prevê a implementação de um sistema de informação para monitoramento das metas.
Programa Nacional de Combate à Obesidade[10]	Programa	O Programa foi aprovado por despacho ministerial em 28 de janeiro de 2005. O objetivo do Programa foi contribuir para a redução do peso nas pessoas com obesidade e nas pessoas que tenham particular risco para desenvolver obesidade. Estratégia voltada para abordagem secundária e terciaria da atenção à saúde. Extinto em 5 de setembro de 2008.
Programa Nacional de Saúde Escolar [Despacho nº12.045/2006][11]	Circular Normativa	O Programa Nacional de Saúde Escolar (PNSE) tem como finalidades: promover e proteger a saúde e prevenir a doença na comunidade educativa; apoiar a inclusão escolar de crianças com Necessidades de Saúde e Educativas Especiais; promover um ambiente escolar seguro e saudável; reforçar os fatores de proteção relacionados com os estilos de vida saudáveis; contribuir para o desenvolvimento dos princípios das escolas promotoras da saúde. As atividades propostas no PNSE estão assentes em dois eixos: a vigilância e proteção da saúde e a aquisição de conhecimentos, capacidades e competências em promoção da saúde.
LIVRO BRANCO SOBRE Uma estratégia para a Europa em matéria de problemas de saúde ligados à nutrição, ao excesso de peso e à obesidade[12]	Política	O documento apresenta uma estratégia para lidar com os problemas de saúde relacionados à nutrição, ao excesso de peso e a obesidade. A estratégia tem como princípio a integração de todas as políticas. Em termos de vigilância, a estratégia indica para o desenvolvimento de sistemas de monitoramento harmonizados entre os países da Comunidade Europeia.
Plataforma Contra a Obesidade[13]	Política	A Plataforma Contra a Obesidade teve como finalidade diminuir a incidência e a prevalência da pré-obesidade e da obesidade através da adoção de medidas integradas de prevenção primária, prevenção secundária e prevenção terciária. No âmbito da prevenção primária, no eixo medidas intersetoriais é destacado como estratégia a realização do II Inquérito Alimentar Nacional. Na atenção secundária, o documento indica a realização de inquéritos epidemiológicos regularmente e de avaliação antropométrica. A Plataforma esteve ativa entre 2007 e 2011 e contou com financiamento privado.
WHO European Action Plan for Food and Nutrition Policy 2007-2012[14]	Plano de Ação	O documento apresenta o segundo plano de ação em termos de política alimentar e nutricional para a Europa. O plano estabelece metas para a saúde, nutrição, segurança dos alimentos e segurança alimentar e nutricional e provê um conjunto coerente de ações integradas para que os Estados Membros considerem em suas políticas nacionais e em seus sistemas de saúde. O documento descreve como área estratégica o monitoramento e

		avaliação dos progressos e desfechos das políticas e programas. A proposta é de que os sistemas de vigilância sejam simples e sustentáveis e que colete dados relativos a estado nutricional, disponibilidade e consumo alimentar, padrões de atividade física, doenças transmitidas por alimentos e outros contaminantes.
Proposta para a Política Nacional de Alimentação e Nutrição[15]	Relatório	O documento apresenta uma proposta para uma Política Nacional de Alimentação e Nutrição. No âmbito da vigilância alimentar e nutricional, o documento propõe a criação de um Observatório Nacional do Consumo Alimentar e do Estado Nutricional, com o propósito de obter informação para a definição e implementação de políticas e/ou programas de alimentação e nutrição.
Action Plan for Implementation of the European Strategy for the Prevention and Control of Noncommunicable Diseases 2012-2016[16]	Plano de Ação	O Plano de Ação apresentado no documento foi desenvolvido para lidar com as doenças crônicas não transmissíveis e seu impacto na saúde, expectativa de vida e qualidade de vida. Os princípios que guiam o Plano são: foco em equidade, fortalecimento dos sistemas de saúde, saúde em todas as políticas, abordagem no curso da vida, empoderamento, balanço entre abordagens individuais e populacionais, programas integrados, abordagem para a sociedade-inteira. O documento indica a necessidade de fortalecimento da vigilância, monitoramento, avaliação e pesquisa. Para isso, são necessários o estabelecimento de sistema de vigilância incluindo informação de carga de doenças, fatores de risco, determinantes sociais e população em risco; a colaboração entre pesquisadores e gestores públicos; e o aumento da disponibilidade e uso de dados desagregados por sexo, idade e status socioeconômico.
Avaliação Antropométrica no Adulto -Direção-Geral da Saúde[17]	Orientação	O documento apresenta orientações para realização do exame antropométrico em pessoas adultas.
HEALTH 2020 – A European policy framework and society for health and well-being[18]	Política	*Health 2020* foca em melhorar a saúde para todos e reduzir as iniquidades em saúde. O documento enumera quatro áreas de prioridades para ação política, entre as quais destaca-se fortalecimento de sistemas de saúde centrados nas pessoas, da capacidade da saúde pública e preparo para emergências, vigilância e resposta.
Plano Nacional de Saúde 2012-2016[19]	Política	O Plano Nacional de Saúde tem como visão maximizar os ganhos em saúde através do alinhamento e da integração de esforços sustentados de todos os setores da sociedade e da utilização de estratégias assentes na cidadania, na equidade e no acesso, na qualidade e nas políticas saudáveis. O documento reforça a necessidade da realização da vigilância epidemiológica.
Programa Nacional para a Promoção da Alimentação Saudável[20]	Programa/ Relatórios	O Programa Nacional para a Promoção da Alimentação Saudável (PNPAS) é um dos programas prioritários do Plano Nacional de Saúde desde 2012. O PNPAS visa promover a saúde da população portuguesa por meio da alimentação e apresenta cinco objetivos: aumentar o

		conhecimento sobre o consumo alimentar da população portuguesa, seus determinantes e consequências; modificar a disponibilidade de certos alimentos, nomeadamente em ambiente escolar, de trabalho e em espaços públicos; informar e capacitar para a compra, produção e armazenamento de alimentos saudáveis; identificar e promover ações transversais que incentivem o consumo de alimentos de boa qualidade nutricional de forma articulada e integrada com outros setores; melhorar a qualificação e o modo de atuação dos diferentes profissionais que pela sua atividade, possam influenciar conhecimentos, atitudes e comportamentos na área alimentar. Os relatórios anuais apresentam o diagnóstico nutricional da população, registro de ações realizadas e o planejamento dos próximos ciclos.
Vienna Declaration on Nutrition and Noncommunicable Diseases in the Context of Health 2020[21]	Declaração	A declaração reafirma o compromisso dos Estados Membros para o enfrentamento dos desafios impostos pelas doenças crônicas não transmissíveis. A declaração indica a necessidade de incentivar a vigilância, monitoramento, avaliação e pesquisa do estado nutricional e comportamento das populações. Além de indicar a necessidade de transparência e acessibilidade dos dados para promover novas pesquisas.
European Food and Nutrition Action Plan 2015-2020[22]	Plano de Ação	A intenção do Plano de Ação é reduzir a carga de doenças crônicas não transmissíveis relacionadas com a alimentação, obesidade e outras formas de má nutrição ainda prevalentes na região. Entre os objetivos do Plano destaca-se o incentivo à vigilância, monitoramento, avaliação e pesquisa, com a consolidação e extensão dos sistemas e estudos já existentes, como por exemplo, *Childhood Obesity Surveillance Initiative* (COSI) e o estudo *Health Behaviour in School-aged Children* (HBSC). O Plano estimula a utilização de repositórios como o WHO *European Database on Nutrition, Obesity and Physical Activity* (NOPA). E indica que os dados disponíveis sejam utilizados de maneira apropriada para a tomada de decisão pelos gestores públicos.
Plano Nacional de Saúde: Revisão e Extensão até 2020[23]	Política	O documento revisita e atualiza o Plano Nacional de Saúde 2012-2016. A atualização do Plano visa rever e consolidar algumas das intervenções já implementadas, melhorar a qualidade das respostas existentes e desenvolver ações considerando o atual contexto social e epidemiológico. O documento propõe o reforço dos sistemas de vigilância epidemiológica em relação aos determinantes de saúde e aos fatores de risco. O Plano apresenta um conjunto de indicadores para monitoramento e também metas a serem alcançadas.
Programa Nacional de Saúde Escolar 2015[24]	Norma	O documento atualiza a Circular Normativa de 2006. O Programa Nacional de Saúde do Escolar (PNSE) tem como objetivos: promover estilos de vida

		saudáveis e elevar o nível de literacia para a saúde da comunidade educativa; contribuir para a melhoria da qualidade do ambiente escolar e para a minimização dos riscos para a saúde; promover a saúde, prevenir a doença da comunidade educativa e reduzir o impacto dos problemas de saúde no desempenho escolar dos/as alunos/as; estabelecer parcerias para a qualificação profissional, a investigação e a inovação em promoção e educação para a saúde em meio escolar.
Action Plan for the Prevention and Control of Noncommunicable Diseases in the WHO European Region 2016-2025[25]	Plano de Ação	O documento atualiza o Plano de 2012-2016, considerando novas evidências, os compromissos já existentes e os progressos de cada Estado Membro. O Plano se propõe a apoiar os Estados Membros no cumprimento das metas de reduzir a mortalidade precoce, reduzir a carga de doenças, melhorar a qualidade de vida e tornar a expectativa de vida mais equitativa. Entre as áreas prioritárias elencadas no Plano destaca-se a vigilância, monitoramento, avaliação e pesquisa. Nessa área, o Plano indica a necessidade de harmonização da vigilância entre os países da Europa e a necessidade de melhorar a comunicação entre especialistas e gestores públicos.
Estratégia Integrada para a Promoção da Alimentação Saudável – Despacho nº 11418/2017, 29 de Dezembro[26]	Política	A Estratégia Integrada para a Promoção da Alimentação Saudável (EIPAS) é uma estratégia voltada para o cumprimento dos objetivos do Programa Nacional para a Promoção da Alimentação Saudável (PNPAS). A EIPAS compreende 51 medidas de intervenção distribuídas em quatro eixos: 1) modificar o meio ambiente onde as pessoas escolhem e compram alimentos através da modificação da disponibilidade de alimentos em certos espaços físicos e promoção da reformulação de determinadas categorias de alimentos; 2) melhorar a qualidade e acessibilidade da informação disponível ao consumidor, de modo a informar e capacitar os cidadãos para escolhas alimentares saudáveis; 3) promover e desenvolver a literacia e autonomia para o exercício de escolhas saudáveis pelo consumidor; 4) promover a inovação e o empreendedorismo direcionado à área da promoção da alimentação saudável.
A new interministerial strategy for the promotion of healthy eating in Portugal: implementation and initial results[27]	Artigo científico [relatório]	No âmbito das ações planejadas na EIPAS, as seguintes ações foram implementadas: 1) definição de padrões de disponibilidade de alimentos em todas as instituições públicas, 2) implementação de imposto do açúcar sobre bebidas adoçadas, 3) implementação de acordo voluntário com o setor da indústria de alimentos para a reformulação de alimentos (trabalho em progresso), 4) elaboração de proposta de modelo interpretativo para rotulagem frontal de alimentos, 5) melhoria da qualidade nutricional dos alimentos oferecidos em programas de apoio a grupos de baixa renda, 6) regulação do marketing para alimentos não saudáveis para crianças.

Despacho nº 6556/2018, de 4 de Julho[28]	Despacho	O Despacho determina que em cada instituição do Sistema Nacional de Saúde (SNS) deverá existir um núcleo/unidade/serviço de Nutrição. O núcleo/unidade/serviço de Nutrição tem por missão desenvolver funções de análise, diagnóstico nutricional, orientação nutricional, intervenção nutricional e monitoramento da alimentação e nutrição, quanto à sua adequação, qualidade, segurança e sustentabilidade, em indivíduos ou em grupos, na comunidade ou em instituições.
Programa Nacional para a Promoção da Alimentação Saudável 2022-2030 [Documento em consulta pública][29]	Programa	O documento propõe a atualização das linhas diretivas do PNPAS para o período de 2022-2030. O PNPAS 2022-2030 visa promover o estado de saúde da população portuguesa, atuando no determinante alimentação com intervenção em três níveis: ambiental, individual e do sistema de saúde. Os valores e princípios do PNPAS 2022-2030 são: independência, transparência e *accountability* (prestação de contas), equidade, colaboração e cooperação, prevenção, rigor científico, evolução e adaptabilidade. A estratégia do PNPAS 2022-2030 é estabelecida em 3 eixos nucleares e complementado por 2 eixos de intervenção transversal. Eixos nucleares: 1) proteger e apoiar, através da criação de ambientes alimentares saudáveis; 2) informar e capacitar, para cidadãos informados e capacitados para escolhas alimentares saudáveis; 3) identificar e cuidar, através do reforço da promoção da alimentação saudável no sistema de saúde e prestação de cuidados de saúde e do acesso a cuidados nutricionais de qualidade. Eixos transversais: 4) monitorar e avaliar, através de um sistema de informação de qualidade em alimentação e nutrição; 5) integrar e articular, para uma ação que coloque a alimentação saudável em todas as políticas e que envolva toda a sociedade. O PNPAS 2022-2030 elenca 13 metas no total, sendo 3 a curto prazo, 8 a médio prazo e 2 a longo prazo.

Referências

1. Portugal. Presidência da República. Decreto-lei no 265/80, de 7 de Agosto [Internet]. ago 7, 1980. Disponível em: https://dre.tretas.org/pdfs/1980/08/07/plain-19170.pdf

2. Portugal. Presidência da República. Decreto-lei no 278/84, de 10 de Agosto [Internet]. 1984. Disponível em: https://dre.tretas.org/pdfs/1984/08/10/plain-14991.pdf

3. Conselho da Comunidade Europeia. Tratado da União Europeia - Tratado de Maastricht [Internet]. 1992 [citado 4 de setembro de 2022]. Disponível em: https://eur-lex.europa.eu/legal-content/PT/TXT/PDF/?uri=CELEX:11992M/TXT&from=PT

4. Conselho da Comunidade Europeia. Tratado da União Europeia [alterações introduzidas pelo Tratado de Amsterdã] [Internet]. 1997 [citado 4 de setembro de 2022]. Disponível em: https://eur-lex.europa.eu/legal-content/PT/TXT/PDF/?uri=CELEX:11997M/TXT&from=PT

5. University of Crete. School of Medicine. Core Report Eurodiet - Nutrition & Diet for Healthy Lifestyles in Europe: Science & Policy Implications [Internet]. European Commission; 2000 [citado 22 de fevereiro de 2022] p. 21. Disponível em: https://ec.europa.eu/health/archive/ph_determinants/life_style/nutrition/report01_en.pdf

6. World Health Organization. The First Action Plan for Food and Nutrition Policy (2000-2005) [Internet]. 2001 [citado 4 de setembro de 2022] p. 45. Disponível em: https://www.euro.who.int/__data/assets/pdf_file/0013/120244/E72199.pdf

7. World Health Organization. Food and nutrition action plans in the WHO European Region - Past, present and future [Internet]. 2003 [citado 4 de setembro de 2022] p. 96. Disponível em: https://apps.who.int/iris/bitstream/handle/10665/107492/E79888.pdf?sequence=1&isAllowed=y

8. Portugal. Ministério da Saúde. Plano Nacional de Saúde: Prioridades para 2004-2010 [Internet]. Portugal: Ministério da Saúde; 2004 [citado 4 de setembro de 2022] p. 36. Disponível em: http://1nj5ms2lli5hdggbe3mm7ms5-wpengine.netdna-ssl.com/files/2015/08/Volume-1-Prioridades.pdf

9. Portugal. Ministério da Saúde. Programa Nacional de Intervenção Integrada sobre Determinantes de Saúde Relacionados com os Estilos de Vida [Internet]. Portugal: Ministério da Saúde; 2004 [citado 4 de setembro de 2022] p. 14. Disponível em: https://www.dgs.pt/documentos-e-publicacoes/estilos-de-vida-saudaveis-pdf.aspx

10. Portugal. Direção-Geral da Saúde. Divisão de Doenças Genéticas, Crónicas e Geriátricas. Programa Nacional de Combate à Obesidade [Internet]. Lisboa: Direção-Geral da Saúde; 2005 [citado 4 de setembro de 2022] p. 24. Disponível em: https://www.dgs.pt/areas-em-destaque/plano-nacional-de-saude/programas-nacionais/programa-nacional-de-combate-a-obesidade-pdf.aspx

11. Portugal. Ministério da Saúde. Direção-Geral da Saúde. Programa Nacional de Saúde Escolar [Internet]. Ministério da Saúde; 2006 jun [citado 21 de fevereiro de 2022] p. 29. Disponível em: https://www.dgs.pt/directrizes-da-dgs/normas-e-circulares-normativas/circular-normativa-n-07dse-de-29062006-pdf.aspx

12. Comissão das Comunidades Europeias. LIVRO BRANCO SOBRE Uma estratégia para a Europa em matéria de problemas de saúde ligados à nutrição, ao excesso de peso e à obesidade [Internet]. Bruxelas; 2007 maio [citado 4 de setembro de 2022] p. 13. Disponível em: https://eur-lex.europa.eu/LexUriServ/LexUriServ.do?uri=COM:2007:0279:FIN:PT:PDF

13. Portugal. Ministério da Saúde. Direção-Geral da Saúde. Plataforma contra a obesidade [Internet]. Ministério da Saúde; 2007 [citado 21 de fevereiro de 2022] p. 22. Disponível em: https://www.dgs.pt/documentos-e-publicacoes/plataforma-contra-a-obesidade-pdf.aspx

14. World Health Organization. WHO European Action Plan for Food and Nutrition Policy 2007-2012 [Internet]. Copenhagen: WHO Regional Office for Europe; 2008 [citado 4 de setembro de 2022] p. 35. Disponível em: https://www.euro.who.int/__data/assets/pdf_file/0017/74402/E91153.pdf

15. Graça P, Gregório MJ. Proposta para a Política Nacional de Alimentação e Nutrição. Direção-Geral da Saúde; 2011 p. 18.

16. World Health Organization. Action Plan for Implementation of the European Strategy for the Prevention and Control of Noncommunicable Diseases 2012-2016 [Internet]. Copenhagen: WHO Regional Office for Europe; 2012 [citado 4 de setembro de 2022] p. 33. Disponível em: https://www.euro.who.int/__data/assets/pdf_file/0019/170155/e96638.pdf

17. Portugal. Direção-Geral da Saúde. Avaliação Antropométrica no Adulto [Internet]. Portugal: Direção-Geral da Saúde; 2013 dez [citado 4 de setembro de 2022] p. 9. Disponível em: https://www.dgs.pt/directrizes-da-dgs/orientacoes-e-circulares-informativas/orientacao-n-0172013-de-05122013-pdf.aspx

18. World Health Organization. HEALTH 2020 - A European policy framework and society for health and well-being [Internet]. Copenhagen: WHO Regional Office for Europe; 2013 [citado 4 de setembro de 2022] p. 22. Disponível em: https://www.euro.who.int/__data/assets/pdf_file/0006/199536/Health2020-Short.pdf

19. Portugal. Ministério da Saúde. Direção-Geral da Saúde. Plano Nacional de Saúde 2012-2016 [Internet]. Portugal: Ministério da Saúde; 2013 [citado 4 de setembro de 2022] p. 111. Disponível em: http://pns.dgs.pt/files/2010/07/Documento-Estrategico-PNS-2011-20161.pdf

20. Portugal. Ministério da Saúde. Direção-Geral da Saúde. Programa Nacional para a Promoção da Alimentação Saudável [Internet]. Lisboa: Ministério da Saúde; 2013 [citado 21 de fevereiro de 2022]. Disponível em: https://alimentacaosaudavel.dgs.pt/alimentacao-em-numeros/relatorios-anuais-pnpas/

21. World Health Organization. Vienna Declaration on Nutrition and Noncommunicable Diseases in the Context of Health 2020 [Internet]. Viena: WHO Regional Office for Europe; 2013 [citado 4 de setembro de 2022] p. 6. Disponível em: https://www.euro.who.int/__data/assets/pdf_file/0003/234381/Vienna-Declaration-on-Nutrition-and-Noncommunicable-Diseases-in-the-Context-of-Health-2020-Eng.pdf

22. WHO Regional Office for Europe. European Food and Nutrition Action Plan 2015-2020 [Internet]. Copenhagen: World Health Organization; 2015 [citado 23 de fevereiro de 2022]. 31 p. Disponível em: https://www.euro.who.int/__data/assets/pdf_file/0003/294474/European-Food-Nutrition-Action-Plan-20152020-en.pdf

23. Portugal. Ministério da Saúde. Direção-Geral da Saúde. Plano Nacional de Saúde: Revisão e Extensão até 2020 [Internet]. Portugal: Ministério da Saúde; 2015 [citado 4 de setembro de 2022] p. 39. Disponível em: http://pns.dgs.pt/files/2015/06/Plano-Nacional-de-Saude-Revisao-e-Extensao-a-2020.pdf.pdf

24. Portugal. Direção-Geral da Saúde. Programa Nacional de Saúde Escolar 2015 [Internet]. Portugal: Direção-Geral da Saúde; 2015 ago [citado 4 de setembro de 2022] p. 110. Disponível em: http://www.arsnorte.min-saude.pt/wp-content/uploads/sites/3/2018/01/Programa_NSE_2015.pdf

25. World Health Organization. Action Plan for the Prevention and Control of Noncommunicable Diseases in the WHO European Region 2016-2025 [Internet]. Copenhagen: WHO Regional Office for Europe; 2016 [citado 4 de setembro de 2022] p. 39. Disponível em: https://www.euro.who.int/__data/assets/pdf_file/0008/346328/NCD-ActionPlan-GB.pdf

26. Portugal. Estratégia Integrada para a Promoção da Alimentação Saudável - Despacho no 11418/2017, 29 de Dezembro [Internet]. Portugal; 2017 [citado 4 de setembro de 2022] p. 4. Disponível em: https://files.dre.pt/2s/2017/12/249000000/2959529598.pdf

27. Graça P, Gregório MJ, de Sousa SM, Brás S, Penedo T, Carvalho T, et al. A new interministerial strategy for the promotion of healthy eating in Portugal: implementation and initial results. Health Res Policy Syst. 30 de outubro de 2018;16(1):102.

28. Portugal. Ministério da Saúde. Despacho no 6556/2018, de 4 de Julho [Internet]. 6556 jul 4, 2018. Disponível em: https://files.dre.pt/2s/2018/07/127000000/1856018561.pdf

29. Portugal. Ministério da Saúde. Direção-Geral da Saúde. Programa Nacional para a Promoção da Alimentação Saudável 2022-2030 [Internet]. Direção-Geral da Saúde; 2022 [citado 20 de fevereiro de 2023]. Disponível em: https://nutrimento.pt/activeapp/wp-content/uploads/2022/10/PNPAS2022_2030_VF.pdf

Quadro S4 Fontes de dados disponíveis para a vigilância alimentar e nutricional em Portugal, 1963 a 2022.

FONTE DE DADOS	OBJETIVOS	ABRANGÊNCIA	TAMANHO DE AMOSTRA	DADOS DISPONÍVEIS: ANTROPOMETRIA, CONSUMO ALIMENTAR, DISPONIBILIDADE ALIMENTAR E/OU SEGURANÇA ALIMENTAR E NUTRICIONAL
Balança Alimentar Portuguesa 1963-1975[1]	O conhecimento das Balanças Alimentares ao longo de uma série de anos permite descrever a evolução das disponibilidades alimentares da população.	Continente	Não se aplica	Disponibilidade alimentar
Inquérito às Receitas e Despesas Familiares 1967-1968[2]	Estimar as despesas de consumo particulares e estabelecer, pelo conhecimento dos bens e serviços efetivamente consumidos pelas famílias observadas, um sistema de ponderação necessário à montagem de um índice de preços no consumidor, ao nível do Continente.	Continente	n=10517 (agregado familiar)	Disponibilidade alimentar
Inquérito Alimentar Nacional 1980[3]	Recolher informação sobre o consumo alimentar.	18 distritos do Continente	Zona urbana (n=1665 famílias) e zona rural (n=4335 famílias). Amostra (n=18000 indivíduos)	Antropometria e consumo alimentar
Inquérito às Receitas e Despesas Familiares 1980-1981[4]	Atualização do índice de preços no consumidor, análise da repartição pessoal do rendimento, análise dos comportamentos de consumo, lançamento de bases para o apuramento direto do consumo final dos agregados, regime de ocupação da habitação, levantamento dos fluxos de poupança.	Continente, Região Autônoma da Madeira e Ilha de São Miguel (Região Autônoma dos Açores)	Continente (n=8040), Açores (n=990), Madeira (n=1152) (agregado familiar)	Disponibilidade alimentar
Balança Alimentar Portuguesa 1980-1992[5]	Acompanhar a evolução da estrutura do padrão alimentar no país e particularmente o impacto na alimentação, decorrente da maior quantidade e diversidade de produtos alimentares disponíveis após a adesão de Portugal à União Europeia.	Nacional	Não se aplica	Disponibilidade alimentar
Inquérito Nacional de Saúde - INS 1987[6]	Descrever o estado de saúde, utilização de cuidados de saúde e determinantes de saúde da população portuguesa residente em unidades	Continente	n=17914 (unidade de alojamento)	Consumo alimentar

	de alojamento familiar.			
Inquérito aos Orçamentos Familiares 1989-1990[7]	Revelar a estrutura e o nível de "consumo" dos agregados familiares.	Nacional	Continente (n=9561), Açores (n=1494), Madeira (n=1205) (unidade de alojamento)	Disponibilidade alimentar
Balança Alimentar Portuguesa 1990-1997[8]	Avaliar a disponibilidade, procura e as tendências de consumo; avaliar a importância relativa dos principais alimentos e grupos de alimentos no total do consumo alimentar.	Nacional	Não se aplica	Disponibilidade alimentar
Balança Alimentar Portuguesa 1990-2003[9]	Avaliar a disponibilidade, procura e as tendências de consumo; avaliar a importância relativa dos principais alimentos e grupos de alimentos no total do consumo alimentar.	Nacional	Não se aplica	Disponibilidade alimentar
Inquérito aos Orçamentos Familiares 1994-1995[10]	Revelar a estrutura e o nível de "consumo" dos agregados, segundo determinadas variáveis de caráter demográfico e socioeconômico.	Nacional	n≈10500 (unidade de alojamento)	Disponibilidade alimentar
Inquérito Nacional de Saúde - INS 1995-1996[6]	Descrever o estado de saúde, utilização de cuidados de saúde e determinantes de saúde da população portuguesa residente em unidades de alojamento familiar.	Continente	n=18000 (unidade de alojamento)	Consumo alimentar
Health Behavior in School Children – HBSC[11]	Descrever o bem estar dos jovens, comportamentos de saúde e o seu contexto social.	Continente	1997/98 (n=3721), 2001/02 (n=2940), 2005/06 (n=3919), 2009/10 (n=4036), 2013/14 (n=4989), 2017/18 (n=5839)	Antropometria e consumo alimentar
Inquérito Nacional de Saúde – INS 1998-1999[6]	Descrever o estado de saúde, utilização de cuidados de saúde e determinantes de saúde da população portuguesa residente em unidades de alojamento familiar.	Continente	n=21808 (unidade de alojamento)	Antropometria e consumo alimentar
Inquérito aos Orçamentos Familiares 2000[12]	Atualizar a estrutura das despesas de "consumo" dos agregados familiares e, ainda a obtenção de informação sobre as condições de vida das famílias.	Nacional	n=12098 (unidade de alojamento)	Disponibilidade alimentar
Balança Alimentar Portuguesa 2003-2008[13]	Avaliar a disponibilidade, procura e as tendências de consumo; avaliar a importância relativa dos principais alimentos e grupos de alimentos no total do consumo alimentar.	Nacional	Não se aplica	Disponibilidade alimentar
Inquérito às Despesas das Famílias 2005-2006[14]	Apurar os indicadores sobre a distribuição do rendimento e o nível e estrutura das despesas	Nacional	n=10403 (unidade de alojamento)	Disponibilidade alimentar

	dos agregados familiares residentes em Portugal, e sobre as respectivas condições de conforto.			
Inquérito Nacional de Saúde – INS 2005-2006[15]	Obter indicadores sobre o estado de saúde e fatores que o determinam para a população residente em Portugal.	Nacional	n=19581 (unidade de alojamento)	Antropometria e consumo alimentar
Balança Alimentar Portuguesa 2008-2012[16]	Avaliar a disponibilidade, procura e as tendências de consumo; avaliar a importância relativa dos principais alimentos e grupos de alimentos no total do consumo alimentar.	Nacional	Não se aplica	Disponibilidade alimentar
Childhood Obesity Surveillance Initiative – COSI[17]	Caracterizar o estado nutricional de crianças portuguesas dos 6 aos 8 anos.	Nacional	2008 (n=3765), 2010 (n=4064), 2013 (n=5935), 2016 (n=6745), 2019 (n=7096) 2022 (n=6205)	Antropometria e consumo alimentar
Inquérito às Despesas das Famílias 2010-2011[18]	Atualizar a estrutura das despesas dos agregados familiares residentes em Portugal e a distribuição dos rendimentos.	Nacional	n=9489 (unidade de alojamento)	Disponibilidade alimentar
INFOFAMÍLIA 2011-2014[19]	Contribuir para o conhecimento da situação de Segurança Alimentar dos agregados familiares em Portugal Continental utentes dos cuidados de saúde primários do Serviço Nacional de Saúde (SNS) e de outras situações de saúde potencialmente associadas às condições socioeconômicas, durante um período de crise econômica e social particularmente intensa entre 2011 e 2014.	Continente	2011 (n=1178), 2012 (n=1208), 2013 (n=1382), 2014 (n=1104)	Antropometria e segurança alimentar e nutricional
Balança Alimentar Portuguesa 2012-2016[20]	Avaliar a disponibilidade, procura e as tendências de consumo; avaliar a importância relativa dos principais alimentos e grupos de alimentos no total do consumo alimentar.	Nacional	Não se aplica	Disponibilidade alimentar
Inquérito Nacional de Saúde – INS 2014[21]	Obter indicadores sobre o estado de saúde e fatores que o determinam para a população residente em Portugal.	Nacional	n=18204 (unidade de alojamento)	Antropometria e consumo alimentar
1º Inquérito Nacional de Saúde com Exame Físico – INSEF 2015[22]	Contribuir para melhorar a Saúde Pública e reduzir as desigualdades em saúde na população residente em Portugal, através da produção,	Nacional	n=4911	Antropometria e consumo alimentar

	disponibilização e comunicação de informação epidemiológica de elevada qualidade sobre o estado de saúde e utilização de cuidados de saúde da população portuguesa.			
Inquérito Alimentar Nacional e de Atividade Física - IAN-AF 2015-2016[23]	Recolher informação nacional sobre o consumo alimentar (incluindo a ingestão nutricional e dimensões de segurança e insegurança alimentares) e sobre a atividade física e a sua relação com determinantes em saúde, nomeadamente os socioeconômicos.	Nacional	n=6553	Antropometria, consumo alimentar e segurança alimentar e nutricional
Inquérito às Despesas das Famílias 2015-2016[24]	Atualizar a estrutura das despesas e distribuição dos rendimentos dos agregados familiares residentes em Portugal, e ainda sobre as condições de conforto dos mesmos agregados familiares.	Nacional	n=11398 (unidade de alojamento)	Disponibilidade alimentar
Balança Alimentar Portuguesa 2016-2020[25]	Avaliar a disponibilidade, procura e as tendências de consumo; avaliar a importância relativa dos principais alimentos e grupos de alimentos no total do consumo alimentar.	Nacional	Não se aplica	Disponibilidade alimentar
Inquérito Nacional de Saúde – INS 2019[26]	Conhecer a avaliação que os portugueses fazem do seu estado de saúde; saber quais os cuidados de saúde adotados pelos portugueses; recolher dados sobre o comportamento dos portugueses em aspectos determinantes para o estado de saúde.	Nacional	n=14617 (unidade de alojamento)	Antropometria e consumo alimentar

Referências

1. Campos J da S. Balanças Alimentares - A Balança Alimentar do Continente Português - Período 1963-1975 [Internet]. Instituto Nacional de Estatística; 1977 [citado 21 de fevereiro de 2022] p. 184. Disponível em: https://www.ine.pt/xportal/xmain?xpid=INE&xpgid=ine_publicacoes&PUBLICACOESpub_boui=265129110&PUBLICACOESmodo=2

2. Portugal. Instituto Nacional de Estatística. Inquérito às Receitas e Despesas Familiares 1967-1968 - Volume 1 [Internet]. Portugal: Instituto Nacional de Estatística; 1970 jan [citado 7 de setembro de 2022] p. 66. Disponível em: https://www.ine.pt/xportal/xmain?

xpid=INE&xpgid=ine_publicacoes&PUBLICACOESpub_boui=447872926&PUBLICACOESmodo=2

3. Gregório MJ, Cavalcanti D, Vasconcelos IAL, Padrão P. Políticas de alimentação e nutrição: Brasil e Portugal. Rev Soc Port Ciênc Nutr E Aliment. 2010;16(1):3–14.

4. Portugal. Instituto Nacional de Estatística. Inquérito às Receitas e Despesas Familiares 1980/81 [Internet]. Portugal: Instituto Nacional de Estatística; 1985 [citado 7 de setembro de 2022] p. 312. Disponível em: https://www.ine.pt/xportal/xmain?xpid=INE&xpgid=ine_publicacoes&PUBLICACOESpub_boui=447872396&PUBLICACOESmodo=2

5. Portugal. Instituto Nacional de Estatística. Balança Alimentar Portuguesa 1980-1992 [Internet]. Lisboa: Instituto Nacional de Estatística; 1994 [citado 7 de setembro de 2022] p. 83. (Estudos). Disponível em: https://www.ine.pt/xportal/xmain?xpid=INE&xpgid=ine_publicacoes&PUBLICACOESpub_boui=284872873&PUBLICACOESmodo=2

6. Dias CM. 25 anos de Inquérito Nacional de Saúde em Portugal. Rev Port Saúde Pública. 2009;51–60.

7. Portugal. Instituto Nacional de Estatística. Inquérito aos Orçamentos Familiares 1989/1990 [Internet]. Lisboa: Instituto Nacional de Estatística; 1992 [citado 7 de setembro de 2022] p. 100. Disponível em: https://www.ine.pt/xportal/xmain?xpid=INE&xpgid=ine_publicacoes&PUBLICACOESpub_boui=447866674&PUBLICACOESmodo=2

8. Portugal. Instituto Nacional de Estatística. Balança Alimentar Portuguesa 1990-1997 [Internet]. Lisboa: Instituto Nacional de Estatística; 1999 [citado 7 de setembro de 2022] p. 111. (Estudos). Report No.: 79. Disponível em: https://www.ine.pt/xportal/xmain?xpid=INE&xpgid=ine_publicacoes&PUBLICACOESpub_boui=375238&PUBLICACOESmodo=2

9. Portugal. Instituto Nacional de Estatística. Balança Alimentar Portuguesa 1990-2003 [Internet]. Lisboa: Instituto Nacional de Estatística; 2006 dez [citado 7 de setembro de 2022] p. 6. Disponível em: https://www.ine.pt/xportal/xmain?xpid=INE&xpgid=ine_destaques&DESTAQUESdest_boui=73985&DESTAQUESmodo=2

10. Portugal. Instituto Nacional de Estatística. Inquérito aos Orçamentos Familiares: Resultados 1994-1995 [Internet]. Lisboa: Instituto Nacional de Estatística; 1997 jul [citado 7 de setembro de 2022] p. 226. Disponível em: https://www.ine.pt/xportal/xmain?xpid=INE&xpgid=ine_publicacoes&PUBLICACOESpub_boui=447867829&PUBLICACOESmodo=2

11. Inchley J, Currie D, Budisavljevic S, Torsheim T, Jåstad A, Cosma A. Spotlight on adolescent health and well-being: findings from the 2017/2018 Health Behavior in School-Aged Children (HBSC) in Europe and Canada. International Report. Volume 1. Key Findings [Internet]. Copenhagen: World Health Organization. Regional Office for Europe; 2020 [citado 21 de fevereiro de 2022] p. 72. Disponível em: https://apps.who.int/iris/bitstream/handle/10665/332091/9789289055000-eng.pdf

12. Inquérito aos Orçamentos Familiares 2000: Principais Resultados [Internet]. Lisboa: Instituto Nacional de Estatística; 2002 jun [citado 7 de setembro de 2022] p. 111. (População e

Condições Sociais). Disponível em: https://www.ine.pt/xportal/xmain?xpid=INE&xpgid=ine_publicacoes&PUBLICACOESpub_boui=133669&PUBLICACOESmodo=2

13. Portugal. Instituto Nacional de Estatística. Balança Alimentar Portuguesa 2003-2008 [Internet]. Lisboa: Instituto Nacional de Estatística; 2010 nov [citado 7 de setembro de 2022] p. 12. (Destaque: Informação à Comunicação Social). Disponível em: https://www.ine.pt/xportal/xmain?xpid=INE&xpgid=ine_destaques&DESTAQUESdest_boui=83386467&DESTAQUESmodo=2

14. Portugal. Instituto Nacional de Estatística. Inquérito às Despesas das Famílias - 2005/2006 [Internet]. Lisboa: Instituto Nacional de Estatística; 2008 [citado 7 de setembro de 2022] p. 135. Disponível em: https://www.ine.pt/xportal/xmain?xpid=INE&xpgid=ine_publicacoes&PUBLICACOESpub_boui=26973702&PUBLICACOESmodo=2

15. Portugal. Instituto Nacional de Estatística. Instituto Nacional de Saúde Doutor Ricardo Jorge. Inquérito Nacional de Saúde - 2005/2006 [Internet]. Lisboa: Instituto Nacional de Estatística. Instituto Nacional de Saúde Doutor Ricardo Jorge; 2009 [citado 7 de setembro de 2022] p. 330. Disponível em: https://www.ine.pt/xportal/xmain?xpid=INE&xpgid=ine_publicacoes&PUBLICACOESpub_boui=69365215&PUBLICACOEStema=00&PUBLICACOESmodo=2

16. Portugal. Instituto Nacional de Estatística. Balança Alimentar Portuguesa 2008-2012 [Internet]. Lisboa: Instituto Nacional de Estatística; 2014 abr [citado 7 de setembro de 2022] p. 25. (Destaque: Informação à Comunicação Social). Disponível em: https://www.ine.pt/xportal/xmain?xpid=INE&xpgid=ine_destaques&DESTAQUESdest_boui=209480091&DESTAQUESmodo=2

17. Rito AI, Paixão E, Carvalho MA, Ramos C. Childhood Obesity Surveillance Initiative: COSI Portugal 2008 [Internet]. Lisboa: Instituto Nacional de Saúde Doutor Ricardo Jorge, Direção-Geral da Saúde; 2011 [citado 21 de fevereiro de 2022] p. 54. Disponível em: http://www.ceidss.com/wp-content/uploads/2020/03/COSI-report-2008.pdf

18. Portugal. Instituto Nacional de Estatística. Inquérito às Despesas das Famílias - 2010/2011 [Internet]. Lisboa: Instituto Nacional de Estatística; 2012 [citado 7 de setembro de 2022] p. 99. Disponível em: https://www.ine.pt/xportal/xmain?xpid=INE&xpgid=ine_publicacoes&PUBLICACOESpub_boui=141577698&PUBLICACOESmodo=2

19. Portugal. Direção-Geral da Saúde. Relatório INFOFAMÍLIA 2011-2014: Quatro anos de monitorização da Segurança Alimentar e outras questões de saúde relacionadas com condições socioeconómicas, em agregados familiares portugueses utentes dos cuidados de saúde primários do Serviço Nacional de Saúde [Internet]. Lisboa: Ministério da Saúde; 2017 [citado 7 de setembro de 2022] p. 114. Disponível em: https://alimentacaosaudavel.dgs.pt/activeapp2020/wp-content/uploads/2020/01/Relato%CC%81rio-INFOFAMI%CC%81LIA-2011-2014.pdf

20. Portugal. Instituto Nacional de Estatística. Balança Alimentar Portuguesa 2012-2016 [Internet]. Lisboa: Instituto Nacional de Estatística; 2017 [citado 7 de setembro de 2022] p. 79. Disponível em: https://www.ine.pt/xportal/xmain?xpid=INE&xpgid=ine_publicacoes&PUBLICACOESpub_boui=289818234&PUBLICACOEStema=00&PUBLICACOESmodo=2

21. Portugal. Instituto Nacional de Estatística. Instituto Nacional de Saúde Doutor Ricardo Jorge. Inquérito Nacional de Saúde 2014 [Internet]. Lisboa: Instituto Nacional de Estatística; 2016 [citado 7 de setembro de 2022] p. 310. Disponível em: https://www.ine.pt/xportal/xmain?xpid=INE&xpgid=ine_publicacoes&PUBLICACOESpub_boui=263714091&PUBLICACOEStema=00&PUBLICACOESmodo=2

22. Santos AJ, Gil AP, Kislaya I, Antunes L, Barreto M, Namorado S, et al. 1o Inquérito Nacional de Saúde com Exame Físico - INSEF 2015: Relatório Metodológico [Internet]. Lisboa: Instituto Nacional de Saúde Doutor Ricardo Jorge (INSA, IP); 2016 maio [citado 7 de setembro de 2022] p. 82. Disponível em: http://repositorio.insa.pt/bitstream/10400.18/3832/3/1_INSEF_relatorio_metodologico.pdf

23. Lopes C, Torres D, Oliveira A, Severo A, Alarcão V, Guiomar S, et al. Inquérito Alimentar Nacional e de Atividade Física, IAN-AF 2015-2016: Relatório metodológico [Internet]. Porto: Universidade do Porto; 2017 [citado 14 de dezembro de 2021] p. 82. Disponível em: https://ian-af.up.pt/sites/default/files/IAN-AF%20Relatorio%20Metodol%C3%B3gico.pdf

24. Portugal. Instituto Nacional de Estatística. Inquérito às Despesas das Famílias - 2015/2016 [Internet]. Lisboa: Instituto Nacional de Estatística; 2017 [citado 7 de setembro de 2022] p. 107. Disponível em: https://www.ine.pt/xportal/xmain?xpid=INE&xpgid=ine_publicacoes&PUBLICACOESpub_boui=277098526&PUBLICACOESmodo=2

25. Instituto Nacional de Estatística. Balança Alimentar Portuguesa 2016-2020 [Internet]. Lisboa: Instituto Nacional de Estatística; 2021 [citado 21 de fevereiro de 2022]. 56 p. Disponível em: https://www.ine.pt/xportal/xmain?xpid=INE&xpgid=ine_publicacoes&PUBLICACOESpub_boui=437140067&PUBLICACOESmodo=2

26. Portugal. Instituto Nacional de Saúde. Departamento de Estatísticas Sociais. Inquérito Nacional de Saúde 2019 [Internet]. 2020 out 21 [citado 7 de setembro de 2022]. Disponível em: https://cse.ine.pt/ngt_server/attachfileu.jsp?look_parentBoui=459375331&att_display=n&att_download=y
